# Matrix protein Tenascin-C promotes kidney fibrosis via STAT3 activation in response to tubular injury

**DOI:** 10.1038/s41419-022-05496-z

**Published:** 2022-12-15

**Authors:** Qionghong Xie, Min Zhang, Xiaoyi Mao, Mingyue Xu, Shaojun Liu, Da Shang, Yunyu Xu, Ruiying Chen, Yi Guan, Xinzhong Huang, Roy Zent, Ambra Pozzi, Chuan-Ming Hao

**Affiliations:** 1 Division of Nephrology, Huashan Hospital, Fudan University, Shanghai, China; 2 Division of Nephrology, Affiliated Hospital of Nantong University, Nantong, China; 3 Division of Nephrology, Vanderbilt University Medical Center, Nashville, TN USA; 4 Department of Veterans Affairs, Nashville, TN USA

**Keywords:** Experimental models of disease, Interstitial nephritis

## Abstract

Accumulating evidence indicates that the extracellular matrix (ECM) is not only a consequence of fibrosis, but also contributes to the progression of fibrosis, by creating a profibrotic microenvironment. Tenascin-C (TNC) is an ECM glycoprotein that contains multiple functional domains. We showed that following kidney injury, TNC was markedly induced in fibrotic areas in the kidney from both mouse models and humans with kidney diseases. Genetically deletion of TNC in mice significantly attenuated unilateral ureteral obstruction-induced kidney fibrosis. Further studies showed that TNC promoted the proliferation of kidney interstitial cells via STAT3 activation. TNC-expressing cells in fibrotic kidney were activated fibroblast 2 (Act.Fib2) subpopulation, according to a previously generated single nucleus RNA-seq dataset profiling kidney of mouse UUO model at day 14. To identify and characterize TNC-expressing cells, we generated a TNC-promoter-driven CreER2-IRES-eGFP knock-in mouse line and found that the TNC reporter eGFP was markedly induced in cells around injured tubules that had lost epithelial markers, suggesting TNC was induced in response to epithelium injury. Most of the eGFP-positive cells were both NG2 and PDGFRβ positive. These cells did not carry markers of progenitor cells or macrophages. In conclusion, this study provides strong evidence that matrix protein TNC contributes to kidney fibrosis. TNC pathway may serve as a potential therapeutic target for interstitial fibrosis and the progression of chronic kidney disease.

## Background

Kidney fibrosis is the final common pathway to end-stage renal disease and characterized by extracellular matrix (ECM) accumulation and destruction of normal structure [1, 2]. Accumulating evidence indicates that the ECM is not only a consequence of fibrosis, but also contributes to the progression of fibrosis, by creating a profibrotic microenvironment or a fibrogenic niche [3, 4].

It has been documented that the ECM not only provides physical scaffolds to cells by forming a three-dimensional network, but also regulates many biologic processes including proliferation, migration, differentiation, survival and morphogenesis during development or in certain physiological/pathophysiological conditions [5–7]. Matricellular proteins (or non-structural matrix proteins) are a group of ECM proteins that are characterized by dynamical expressions and regulatory roles [8]. Rather than serving as stable structural elements, they are usually transiently expressed during development or after injury [9]. They contain multiple functional domains, such as binding sites for other ECM proteins, ligands for cell surface receptors, and sites that can sequester specific growth factors, playing important roles in regulating cellular processes [10].

Tenascin-C (TNC), a hexametric glycoprotein, is a member of the matricellular proteins that is expressed during development and is at low levels in most of the adult tissues, but re-induced following injury and associated with the severity of diseases and prognosis [11, 12]. The monomer of TNC contains an N-terminal assembly domain, epidermal growth factor-like (EGF-L) repeats, fibronectin type-III (FNIII) like repeats and a C-terminal fibrinogen-like globe [13, 14]. These functional domains have been suggested to interact with specific cell-surface receptors, such as epidermal growth factor receptor (EGFR), integrins and toll-like receptor 4 (TLR4) [13]. They can also recruit cytokines and growth factors, and present them to or prevent them from cell-surface receptors depending on their relative locations [14]. Mice lacking TNC develop normally, suggesting that TNC is not indispensable for development. However, accumulating evidence show that TNC is markedly increased in the fibrotic tissues and associated with organ fibrosis [15–17]. Recently, TNC was also found to promote renal interstitial fibroblast proliferation via integrin/focal adhesion kinase/mitogen-activated protein kinase pathway [18].

In the present study, we characterized the cells that expressed TNC and examined their role in renal fibrosis. This study provided novel information about the role of TNC in renal fibrosis.

## Methods

### Patients

To characterize the distribution of TNC expression in normal human kidney, specimens from healthy parts of human carcinoma nephrectomy were obtained. TNC mRNA was measured by qPCR in fresh tissue, and the distribution of TNC protein was investigated in paraffin-embedded tissue by immunohistochemistry (IHC). To examine the induction of TNC in diseased kidneys, TNC protein was detected in paraffin-embedded sections from patients with biopsy-proven interstitial fibrosis (IgA nephropathy) by IHC. This study was approved by the ethics committee of Huashan Hospital, Fudan University, and informed consents were signed.

### Animals and Models

#### Mice

TNC^CreER2-eGFP/+^ mouse line was generated as described previously [19]. In brief, an inducible CreER2 gene with an eGFP reporter was knocked into the 2^nd^ exon of TNC at the site of starting codon ATG. The insertion of this cassette also resulted in TNC deletion (Fig. 2A). The TNC deletion (TNC^-/-^) mice were fertile, and developed normally. Male TNC^-/-^ mice and their wild-type littermates, aged 8–12 weeks, were used to examine the role of TNC in fibrosis. R26^tdTomato^ reporter mice were purchased from the Jackson Laboratory (stock number, 007909). The bi-transgenic TNC^CreER2-eGFP/+^;R26^tdTomato/+^ reporter mice were generated by crossing TNC^CreER2-eGFP/+^ with R26^tdTomato^ mice. All of the mice were maintained in the animal facility of Fudan University and allowed free access to standard rodent chow and water. All of the animal experiments were approved by the Institutional Animal Care and Use Committees of Fudan University.

#### TNC reporter mice

In the TNC^CreER2-eGFP/+^ mice, eGFP is driven by endogenous TNC promoter (TNCp-CreER2-IRES-eGFP), and is thus used as a TNC reporter. In the bi-transgenic TNC^CreER2-eGFP/+^;R26^tdTomato/+^ reporter mice, the recombination is induced by TNC-promoter-driven CreER2 in the presence of tamoxifen, and then tdTomato (red fluorescence protein) will label the TNC-expressing cells and their daughter cells permanently.

#### Models

UUO-induced kidney fibrosis model was used in our study. The mice were anesthetized with chloral hydrate (400 mg/kg body weight) by intra-peritoneal injection and body temperatures were maintained at 36.5–37.5 °C throughout the procedure. The left ureter was exposed via a flank incision and ligated with two 3.0 silk ties at the level of the lower renal pole. The mice were sacrificed at day 7, 10, or 14 after the operation. The EdU (Invitrogen, C10339) cooperation assay was used to assess cell proliferation of the kidney: 0.1 mg of EdU was injected (i.p.) at day 3 and 5 after UUO operation and the mice were sacrificed at day 7. Each group for comparison included 6 to 8 mice. The investigators were blinded to the animal when accessing the outcomes.

### Cell experiments

#### Cell lines

The TNC-expressing cells (TNC-Cell) were obtained by sorting the tdTomato-positive cells in the UUO kidneys of TNC^CreER2-eGFP/+^;R26^tdTomato/+^ mice using flow cytometry, and immortalized using SV40 T lentivirus (Fig. 9B). This cell line was cultured in polylysine (20 μg/mL, Sigma, P1399) coated dish with DMEM/F12 media containing 10% FBS, 1 ng/mL basic-FGF (Peprotech, 400-29) and 5 ng/mL insulin (Sigma, I0305000). The normal rat kidney fibroblast NRK49F was purchased from American Type Culture Collection (ATCC). Both NRK49F and mouse embryo fibroblast NIH3T3 were cultured in DMEM media (GIBCO, C11995500BT) containing 10% heat-inactivated fetal bovine serum (FBS, GIBCO, 10270-106), 100U/ml penicillin and 100 μg/ml streptomycin. Mycoplasma contamination was tested by polymerase chain reaction (PCR). Cells were incubated at 37 °C in a 5% CO_2_, humidified atmosphere.

#### Intervention

The cultured cells at 60% confluence were incubated with 1% FBS-containing media for 24 h followed by treatment of human TNC (Millipore, CC605, 5 μg/mL or 10 μg/mL) or TGFβ (2 ng/mL to 10 ng/mL, Sigma, APREST95171). The STAT3 inhibitor Stattic (Selleck, S7024, 2 μM) or EGFR inhibitor Gefitinib (5 μM, Selleck, ZD-1839) were added to the media 30 min before TNC treatment.

#### Cell Proliferation

After TNC treatment for 24 h, the number and viability of the cultured cells were determined using the Cell Counting Kit-8 (CCK-8, Dojindo Laboratories, CK04) following the manufacturer’s instructions. Briefly, water-soluble tetrazolium salt WST-8 is reduced by dehydrogenase in the cells and turns into an orange formazan dye which is soluble in the culture media, and the amount of the formazan dye is proportional to the number of living cells. The cell proliferation was determined by detecting the proportion of EdU-positive cells using the Click-iT® EdU Imaging Kits (Invitrogen, C10339) according to the manufacturer’s instructions. EdU is detected based on a copper-catalyzed covalent reaction between an alkyne (contained in the EdU) and azide (contained in the Alexa Fluor dye).

### Histology and pathology

#### Tissue preparation

The mice were anesthetized with chloral hydrate (500 mg/kg body weight) intra-peritoneal injection before sacrifice and immediately perfused with 60 ml ice-cold PBS via the left ventricle. The kidneys were hemi-sectioned horizontally and fixed in 4% paraformaldehyde (PFA) on ice for 1 hp. Then half of each kidney was incubated in 30% sucrose-PBS at 4 °C overnight and embedded in OCT, and the other half was processed and embedded in paraffin.

#### Immunohistochemistry

Paraffin-embedded 2μm-thick sections of the human kidney were deparaffinized and rehydrated in water. After incubation with 3% H_2_O_2_ for 30 min at room temperature (RT), the tissues were given microwave antigen retrieval in citrate buffer pH 6.0 (100% power for 10 min), blocked with 5% BSA in PBS for 1 h at RT, and followed by the incubation with anti-human TNC antibody (Sigma rabbit polyclonal, HPA004823, 1:500) at 4 °C overnight. After 3 washes with PBS, the samples were incubated in horseradish peroxidase (HRP) conjugated secondary antibody for 45 min at RT, followed by coloration with 3,3- diaminobenzidine solution (DAB, Gene Tech, GK6005).

#### Immunofluorescence

Frozen tissue embedded in OCT was cut into 5 μm thick section. For IF analysis, the sections were fixed in cold acetone for 3 min, washed in PBS, blocked in 5% bovine serum albumin (BSA) for 30 min and then incubated with primary antibodies in blocking buffer overnight at 4 °C. The primary antibodies included: anti-GFP (Abcam rabbit polyclonal, ab290, 1:200; Aves Labs chicken antibodies, GFP-1020, 1:500), anti-AQP2 (Santa Cruz goat polyclonal, sc-9882, 1:100), anti-CD31 (Abcam rat monoclonal, ab56299, 1:100), anti-PDGFRβ (eBioscience rat monoclonal, 14-1402, 1:100), anti-CD34 (Abcam rat monoclonal, ab8158, 1:200), anti-CD44 (Abcam rat monoclonal, ab119863, 1:200), anti-α-SMA (Sigma mouse monoclonal, C6198, 1:100), anti-FSP-1 (Abcam rabbit polyclonal, ab27957, 1:200), anti-NG2 (Millipore rabbit polyclonal, AB5320B, 1:100), anti-TNC (Sigma rat monoclonal, T2551, 1:100), anti-F4/80 (AbD serotec rat monoclonal, MAC497R, 1:200), anti-CD68 (AbD serotec rat monoclonal, MAC497R, 1:200), and anti-THP (Santa Cruz rabbit polyclonal, sc-20631, 1:200) antibody. After 3 washes in PBS, the tissue was incubated with FITC- or Cy3- conjugated anti-IgG/IgY secondary antibody (Millipore, 1:200) for 1 h at room temperature (RT), followed by 3 washes in PBS and then covered by Vectashield Mounting Medium with DAPI (Vector Labs, H-1200-10). Fluorescein lotus tetragonolobus lectin (LTL, Vector Labs, FL-1321-2, 1:100) was used to label proximal tubules.

#### EdU detection

Paraffin-embedded renal tissue from the mice which had received EdU (Invitrogen, C10339) injection after the operation was used for EdU detection according to the manufacturer’s instructions. EdU-positive cells were calculated per high power field to compare the proliferation between wild type and TNC^−/−^ mice.

### Quantitative RT-PCRs

Renal cortex and papilla isolated from mice kidneys were used to measure TNC mRNA and TNC reporter eGFP mRNA to determine the expression of TNC in different regions of kidney. The entire kidney from UUO model was homogenized to exam the TNC expression and evaluate the severity of fibrosis by detecting the mRNA of TNC, collagen Iα, fibronectin and PAI-1. Total mRNA was extracted using TRIzol Reagent (Invitrogen), reversely transcribed using RevertAid RT reagent Kit (Takara) according to the manufacturer’s protocol. Levels of mRNA were determined by real time qPCR using SYBR Premix Ex Taq (Takara) and normalized to the eukaryotic 18 s rRNA or β-actin. The primers used were TNC: F 5′-CAA CTG TGC CCT GTC CTA C-3′, R 5′-AAC GCC CTG ACT GTG GTT A-3′; eGFP: F 5′-CCT CAA GGA CGA CGG CAA C-3′, R 5′-CTC GAT GCG GTT CAC CAG-3′; collagen Iα: F 5′-TGA CTG GAA GAG CGG AGA G-3′, R 5′-GAC GGC TGA GTA GGG AAC A-3′; fibronectin: F 5′-TGG GAG CAT TGT TGT GTC-3′, R 5′-AGC GGT GTC ACT ACT CTG T-3′; PAI-1: F 5′-ACT TTA CCC CTC CGA GAA-3′, R 5′-CCT GCT GAA ACA CTT TTA C-3′.

### Western Blot

Total protein was extracted from the whole kidney or cultured cells using RIPA buffer (0.05 M Tris, 0.15 M NaCl, 1% Triton X-100, 1% sodium deoxycholate, 0.1% SDS, pH7.4) with PMSF, protease inhibitor cocktail and phosphatase inhibitor cocktail (Roche). The concentration of the protein was determined using the BCA protein assay kit (Beyotime). For Western blot, equal amount of protein (20–50 μg per lane) was loaded in a 7.5% or 10% SDS-PAGE mini-gel and transferred to a PVDF membrane (Millipore). The membrane was blocked with 5% BSA in PBST buffer (100 mM PBS, pH 7.5, 0.1% Tween-20) and then incubated in primary antibody diluted with blocking buffer overnight at 4 °C. The primary antibodies included: anti-TNC (IBL, 10337), anti-collagen Iα (Novus, NBP1-30054), anti-αSMA (Sigma, A2066), anti-STAT3 (CST, 12640), anti-phospho-STAT3 (Tyr 705, Αbcam, ab76315), anti-EGFR (abcam, ab52894), anti-phospho-EGFR (TYR1068, CST, 2234), anti-GAPDH (CST, 5174) and anti-β-actin (Proteintech, 66009-1-Ig) antibody. Membranes were then incubated with appropriate secondary antibodies and subjected to chemiluminescence detection using ECL Reagent (Millipore, WBKLS0500).

### Single Nucleus RNA Sequencing Analysis

UUO day 14 single-cell dataset was downloaded from GEO (GSE119531) [20]. The dataset was processed and analyzed using Seurat software [21]. Data was normalized using “LogNormalize” with a scale factor of 10000. The top 2000 most variable genes were identified using “vst” method. The atlas was subjected to scaling using all genes and PCA analysis using the top 2000 most variable genes. The UMAP embedding was obtained using the top 30 principal components. The cells were annotated the same as reported by the original study. To improve visualization, the cellular identity was sorted as following: JGA, Pod, PT(S1), PT(S2), PT(S3), Prolif. PT, Dediff. PT, DL and tAL, TAL, CNT, DCT, CD-PC, IC, EC, Act. Fib1, Act. Fib2, MP.

### Statistical analysis

Statistical analysis was performed using Prism 9.0 (GraphPad Software Inc.). Comparison of two factors was performed by two-tailed *t* test and comparison of two factors with multiple levels was performed by two-way ANOVA test. A *P* value < 0.05 was considered significant. The results were presented as means and error bars indicate ±SEM.

## Results

### TNC was constitutively expressed by the renal medullary stromal cells in normal kidney

**Fig. 1:**
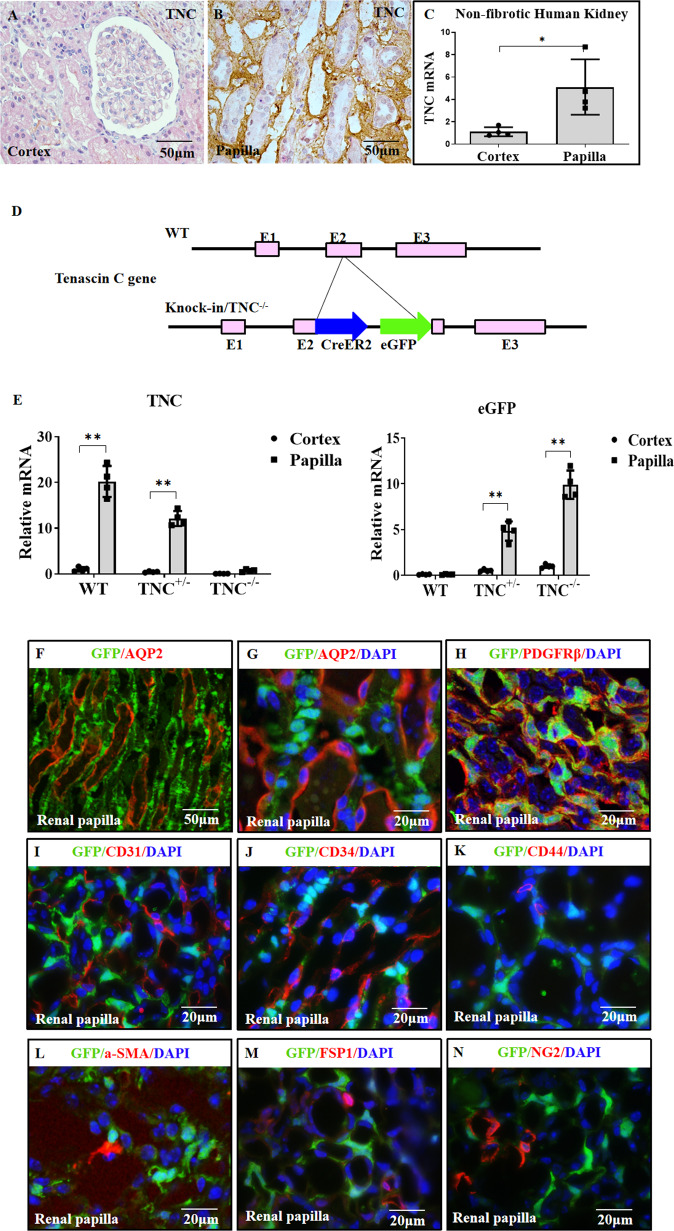
TNC was constitutively expressed by renal medullary stromal cells in normal kidney. In normal human kidney (kidneys resected because of renal carcinoma), TNC was rarely detected in the cortex, but rich in the medullary interstitium (**A**, **B**, *n* = 4, 4 sections for each). Quantitative PCR also showed TNC mRNA was significantly higher in non-fibrotic renal papilla than in cortex (**C**, *n* = 4, *p* = 0.019). To explore the role of TNC, a TNC-promoter-driven inducible CreER2 knock-in mouse line with an eGFP reporter was generated, and the insertion of CreER2-eGFP lead to TNC deletion (**D**). Messenger RNA of TNC and TNC reporter eGFP was significantly higher in the renal papilla than in the renal cortex (**E**, *n* = 4 for each, *p* < 0.01). Co-staining for eGFP and AQP2 showed that eGFP-positive cells were located in the renal medullary interstitium (**F**, **G**). These eGFP-positive cells were PDGFRβ positive, consistent with stromal cells (**H**), but negative for CD31 (a marker of endothelial cells, **I**), CD34 (a marker of endothelial cells and early hematopoietic stem cells and progenitors, **J**) and CD44 (a marker of progenitors, **K**). They were also negative for αSMA (a marker of myofibroblasts, **L**), FSP1 (a marker of fibroblasts, **M**) and NG2 (a marker of pericytes, **N**). (*n* = 4, 3 slides for each).

To determine the distribution of TNC in non-fibrosis kidneys, we examined TNC expression using immunohistochemistry (IHC) in non-fibrosis human kidneys and found that TNC immuno-protein was primarily located in the interstitium of renal medulla, but rarely detected in the renal cortex (Fig. 1A, B). This result was supported by TNC mRNA measurement in human kidneys which were resected because of renal carcinoma (Fig. 1C).

Since TNC is an extracellular matrix protein, IHC will not be able to identify cells that express TNC. Therefore, we developed a TNC-promoter-driven eGFP reporter mice (Fig. 1D) [19]. TNC and eGFP mRNA measurement in wild-type or eGFP reporter mouse kidneys also supported that TNC was predominantly expressed in renal papilla (Fig. 1E). Co-staining for TNC reporter eGFP and AQP2, a marker of collecting ducts, showed that eGFP positive cells were not epithelial cells. They were primarily expressed by the renal medullary interstitial cells (RMICs) in normal mouse kidney (Fig. 1F, G and Supplementary 1F, G) [22, 23]. To characterize the cell types that expressed TNC, we co-stained eGFP with specific cell markers in normal kidney and found that eGFP-positive cells expressed platelet-derived growth factor receptor beta (PDGFRβ), a marker of stromal cells of mesenchymal origin [24] (Fig. 1H and Supplement 1H). They did not express CD31 (a marker of endothelial cells [25]), CD34 (a marker of endothelial cells and early hematopoietic stem cells and progenitors [26]), or CD44 (a marker of progenitors [27, 28]), suggesting that TNC was neither expressed in endothelial cells nor progenitor cells (Fig. 1I–K and Supplementary 1I–K). In the normal renal medulla, there were only a few cells positive for αSMA (a marker of myofibroblasts [29]), FSP1 (recognized as a specific marker of fibroblasts [30], but subsequently identified as a marker of macrophages [31]), or NG2 (a transmembrane glycoprotein used to label pericytes [32]). We did not find eGFP was colocalized with these three markers (Fig. 1L–N and Supplementary Fig. 1L–N).

### TNC was markedly induced in the fibrotic area of diseased kidney

**Fig. 2:**
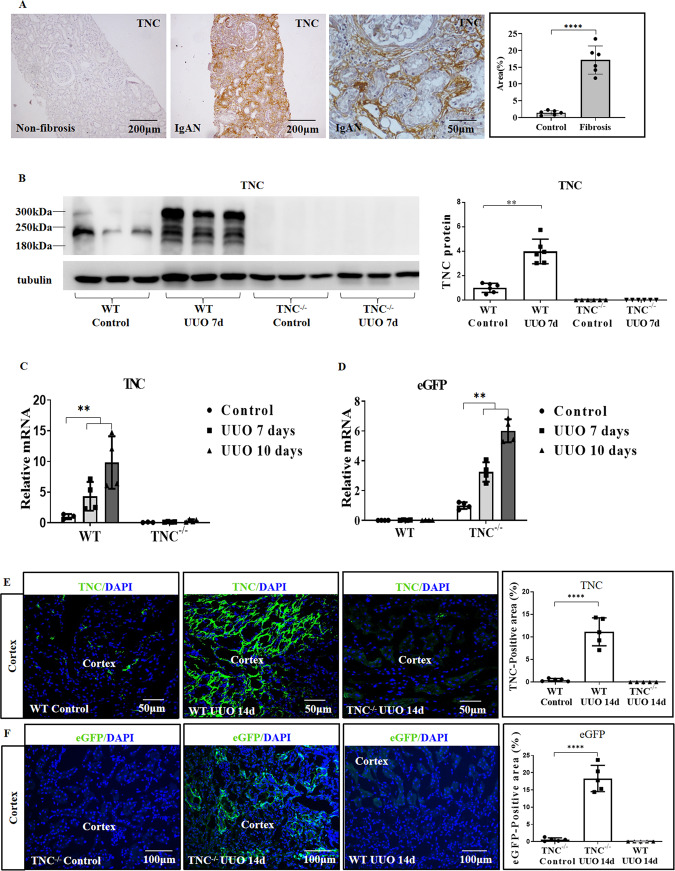
TNC was significantly increased in the fibrotic kidneys. In patients, TNC level was low in renal cortex of non-fibrotic kidney with minimal change disease, and significantly increased in fibrotic kidneys with IgA nephropathy (**A**, *n* = 6, *p* < 0.0001). In UUO induced kidney fibrosis mouse model, TNC protein was dramatically increased with different splicing variants (**B**, *n* = 6, *p* < 0.01), and TNC mRNA in wild-type mice and eGFP mRNA in eGFP reporter mice were also significantly elevated (**C**, **D**, *n* = 4 for each time point *p* < 0.01). IF showed that TNC was markedly increased in renal cortex, and eGFP staining in the reporter mice confirmed this result (**E**, **F**, *n* = 5, *p* < 0.0001).

We previously reported that TNC was significantly increased in injured glomeruli of IgA nephropathy and expressed by PDGFRβ positive mesangial cells [33]. TNC was also markedly induced in the fibrotic interstitial area of the renal cortex of biopsy specimens from patients with IgA nephropathy or from patients with diabetic nephropathy (Fig. 2A). To further examine the expression of TNC in kidney fibrosis, we generated a unilateral ureteral obstruction (UUO) mouse model and found that TNC protein and mRNA were significantly increased in the fibrotic kidneys (Fig. 2B, C, E). Transgenic TNC reporter mice also showed a remarkable increase of eGFP mRNA expression and eGFP positive cells in the UUO kidneys (Fig. 2D, F). Similar results were also found in the ischemia reperfusion (IR) induced kidney fibrosis model (Supplementary Fig. 2).

### TNC deficiency reduced kidney fibrosis in animal models

**Fig. 3:**
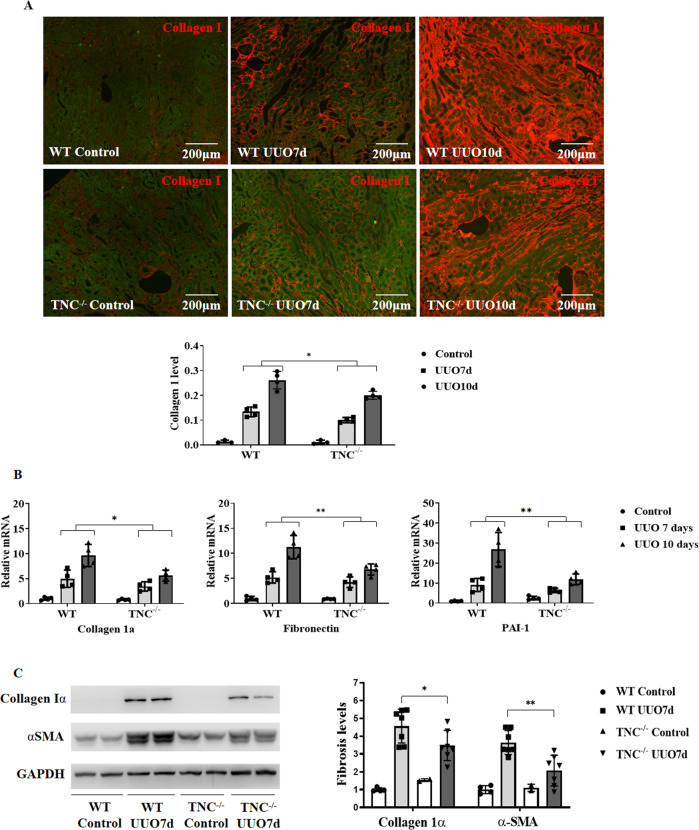
TNC deficiency reduced kidney fibrosis in animal models. Deletion of TNC (TNC^−/−^) significantly attenuated the induction of collagen I assessed by IF following UUO by approximate 30% at day 7 and 10 compared with their wild-type littermates (**A**, *n* = 4 for each time point, two-way ANOVA *p* < 0.05). Consistently, the expression of fibrosis markers, such as collagen Iα, fibronectin and plasminogen activator inhibitor-1 (PAI-1), were significantly lower in TNC^−/−^ mice at UUO day 7 and 10 (**B**, *n* = 4 for each time point, two-way ANOVA, *p* < 0.05). Western blot showed that the proteins of collagen Iα and α-SMA were also significantly reduced in TNC^−/−^ mice at UUO day 7 (**C**, *n* = 7, *p* < 0.05).

To investigate the role of TNC induction in kidney fibrosis, we generated a TNC homozygous knockout mice. Deletion of TNC (TNC^−/−^) significantly attenuated the induction of collagen I expression in the kidneys assessed by IF following UUO by approximate 30% at day 7 and 10 compared with their wild-type littermates (Fig. 3A, *p* < 0.05). Consistently, mRNAs of fibrosis markers, such as collagen Iα, fibronectin and plasminogen activator inhibitor-1 (PAI-1), were significantly lower in the TNC^−/−^ mice after UUO at day 7 and 10 (Fig. 3B, *p* < 0.05). Western blot showed that the proteins of collagen Iα and α-SMA were also significantly reduced in TNC^−/−^ mice after UUO at day 7 (Fig. 3C, *n* = 7, *p* < 0.05).

### TNC enhanced fibrosis by promoting fibroblasts proliferation via STAT3 pathway

**Fig. 4:**
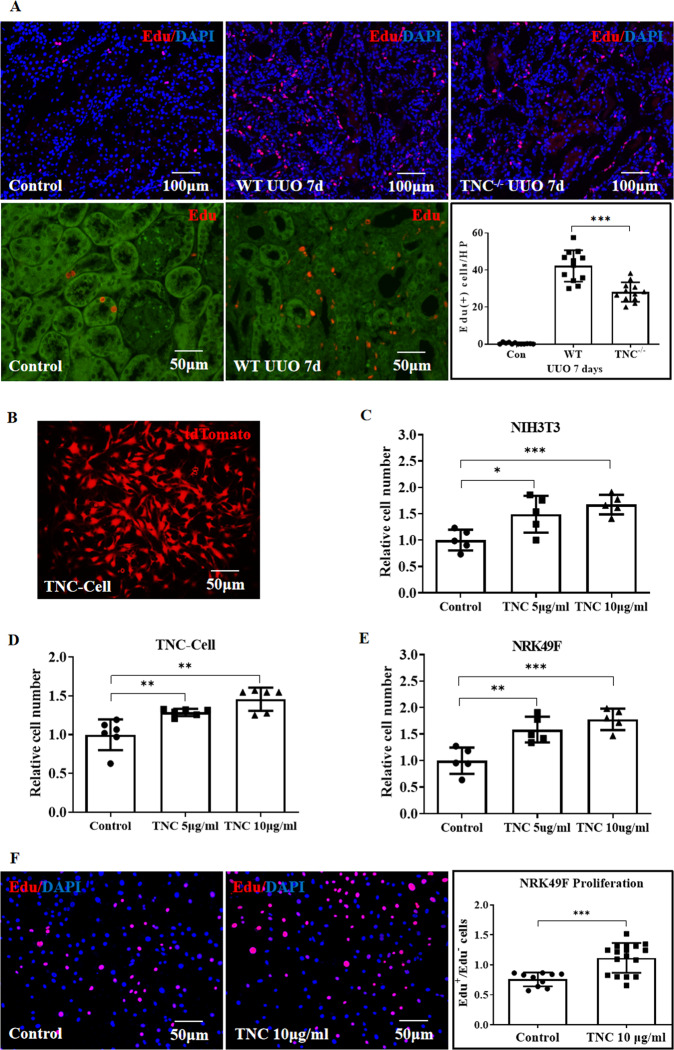
TNC promoted fibroblasts proliferation. In vivo, EdU positive cells were markedly increased and predominantly located in the renal interstitium after UUO, and TNC deletion significantly reduced the number of EdU-positive cells in the obstructed kidney compared with wild-type mouse (**A**, *n* = 4 mice, 3 slides for each mouse, *p* < 0.05). To further examine the effect of TNC on cell proliferation, TNC-expressing cells (TNC-Cell) were obtained by sorting the tdTomato-positive cells in UUO kidneys, and then immortalized by transfecting SV40 T lentivirus (**B**). Exogenous TNC dose dependently increased the cell number of TNC-Cell, NIH3T3 and NRK49F in vitro, assessed by CCK8 kit (**C**–**E**, *p* < 0.01). Exogenous TNC also markedly increased EdU incorporation in NRK49F cells, consistent with promoting cell proliferation (**F**, *p* < 0.001).

To explore the mechanism by which TNC enhanced fibrosis, we examined the effect of TNC on interstitial cell proliferation and the potential signaling mechanism. Firstly, we evaluated the cell proliferation using EdU incorporation study. In vivo, EdU-positive cells were markedly increased and predominantly located in the renal interstitium after UUO, and TNC deletion significantly reduced the number of EdU-positive cells in the obstructed kidney compared with wild-type mouse (Fig. 4A). To further examine the effect of TNC on cell proliferation, we cultured TNC-Cell (an interstitial cell line obtained from fibrotic kidney, Fig. 4B), NIH3T3 and NRK49F cells. Exogenous TNC dose-dependently increased the number of these cultured cells as assessed by CCK8 kit (Fig. 4C–E). Exogenous TNC also markedly increased EdU incorporation in NRK49F cells, consistent with increasing cell proliferation (Fig. 4F).

**Fig. 5:**
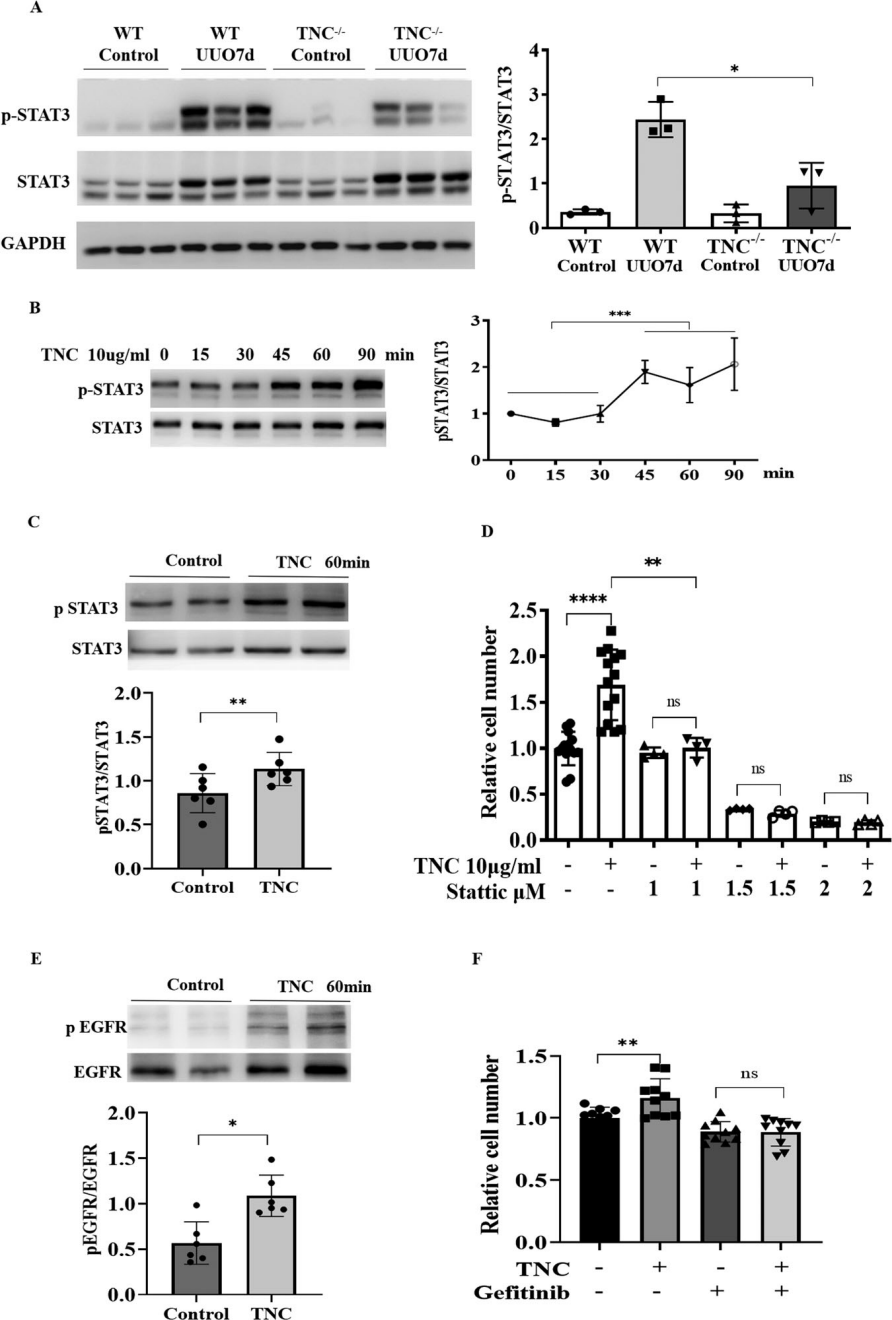
TNC promoted stromal cells proliferation via STAT3 pathway. In vivo, STAT3 and phospho-STAT3 were markedly increased after UUO, and TNC deletion significantly reduced the phospho-STAT3 levels (**A**). In cultured fibroblasts, exogenous TNC markedly increased the phosphorylation of STAT3, peaking at 45 min (**B**, **C**). The effect of TNC on cell proliferation was blocked by the STAT3 inhibitor Stattic (**D**). STAT3 is a downstream target of epidermal growth factor receptor (EGFR). TNC also increased the phosphorylation of EGFR (**E**), and EGFR inhibitor reduced the cell number of TNC-induced fibroblasts proliferation (**F**).

To determine the signaling mechanism by which TNC-promoted cell proliferation, we screened 45 phospho-kinases in cultured fibroblast treated with or without TNC, and identified STAT3 as one of the candidates that responded to TNC treatment. The potential significance of STAT3 signaling in mediating the effect of TNC was further validated in UUO kidney and cultured cells. In vivo, the STAT3 and phospho-STAT3 levels were markedly increased after UUO, and TNC deletion significantly reduced the phospho-STAT3 levels (Fig. 5A). In cultured cells fibroblasts, TNC markedly increased the phosphorylation of STAT3, peaking at 45 min (Fig. 5B, C). The effect of TNC on cell proliferation was blocked by the STAT3 inhibitor Stattic (Fig. 5D). It has been well documented that STAT3 is a downstream target of the epidermal growth factor receptor (EGFR). TNC also increased the phosphorylation of EGFR (Fig. 5E), and inhibitor of EGFR reduced the cell number of TNC-induced fibroblasts proliferation (Fig. 5F).

### TNC-expressing cells in fibrotic kidneys surrounded the injured tubules

**Fig. 6:**
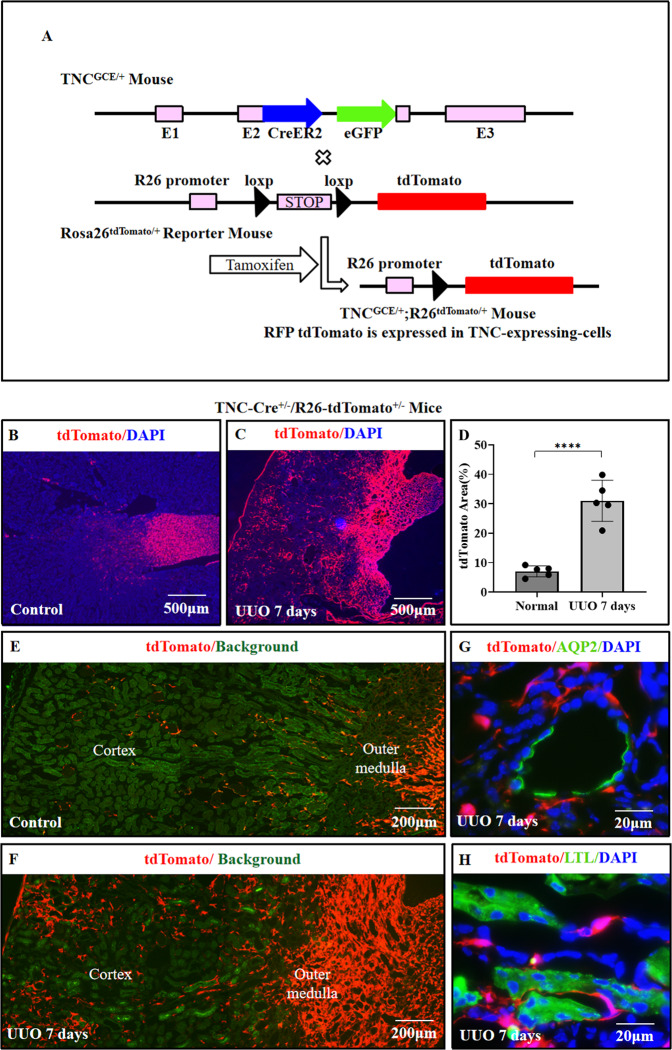
TNC-expressing cells were significantly increased in fibrotic kidneys. In bi-transgenic TNC^CreER2-eGFP/+^; R26^tdTomato/+^ mice, the recombination happens in the cells that express TNC in the presence of tamoxifen, and then these TNC-expressing cells and their daughter cells will be labeled with red fluorescence protein tdTomato permanently (**A**). In normal kidneys, tdTomato also identified TNC-expressing cells rich in the papilla, scattered in the outer medulla, and rare in the cortex (**B**, **E**). After UUO (tamoxifen given for 5 days after operation), the tdTomato positive cells were significantly increased in both the renal cortex and medulla (**C**, **D**, **F**, *p* < 0.0001), and these cells were neither AQP2 nor Lotus Tetragonolobus Lectin (LTL) positive tubular epithelial cells (**G**, **H**). (*n* = 5, 3 slides for each).

To confirm the above results that TNC-expressing cells were significantly induced in fibrosis and explore their characterization, we generated another TNC mouse reporter TNC^CreER2-eGFP/+^;R26^tdTomato/+^, in which the cells that express TNC will be labeled with red fluorescence protein tdTomato permanently following tamoxifen (Fig. 6A). There are some differences between tdTomato reporter mice and eGFP reporter mice. First, the tdTomato reporter not only labels the cells which are expressing TNC but also the cells once expressed TNC, while the eGFP reporter only labels the cells which are expressing TNC. Second, tdTomato is only expressed in the presence of tamoxifen while eGFP not. Third, the expression of tdTomato is driven by the promoter Rosa26 and the expression level is high with strong autofluorescence. The tdTomato expression does not reflect TNC expression, while eGFP does. Fourth, the downstream IRES-eGFP protein may be less efficiently translated than the coding sequence of Cre which is directly adjacent to the promoter. However, tamoxifen induction efficiency as well as excision rate vary among tissue types.

Using this bi-transgenic reporter mice TNC^CreER2-eGFP/+^;R26^tdTomato/+^, we confirmed that TNC-expressing cells were primarily expressed in the medulla of normal kidney (Fig. 6B, E). Then we generated UUO model in this mice and administered tamoxifen right after operation, and found that the tdTomato positive cells were significantly increased in the cortex of fibrotic kidneys (Fig. 6C, D, F). These tdTomato-positive cells were neither stained for Lotus tetragonolobus lectin (LTL, proximal tubule) nor expressed AQP2 (collecting duct), suggesting that they were not epithelial cells (Fig. 6G, H and Supplementary Fig. 3).

**Fig. 7:**
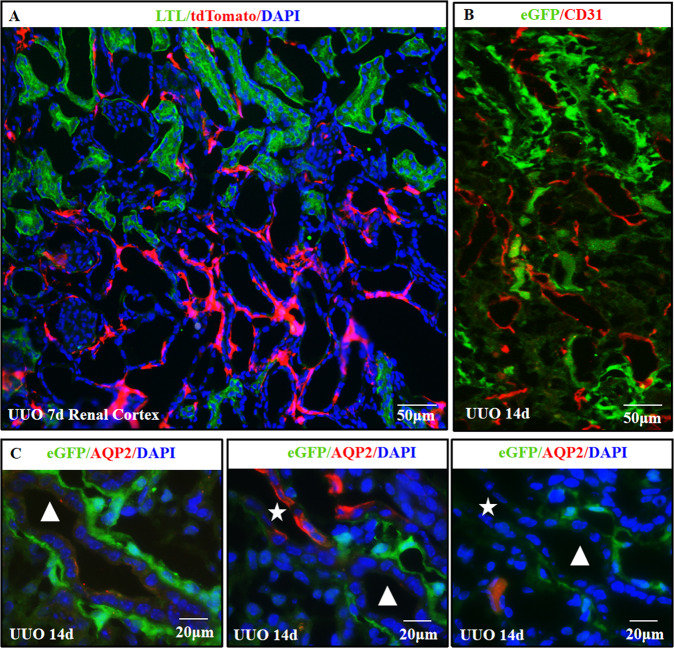
TNC-expressing cells surrounded the injured renal tubules in fibrotic kidneys. TNC reporter eGFP or tdTomato-positive cells were predominantly located adjacent to the tubular structures in UUO kidneys (**A**, **B**). These tubules were negative for CD31, a maker of endothelial cells, suggesting not vessels (**B**). They were dilated and negative for epithelial markers including LTL and AQP2, presumably injured tubules which lost their markers (**A**–**C**). Serial section experiments showed that these tubules (△) had continuation to structures with positive AQP2 staining(☆), further supporting that TNC-expressing cells were localized surrounding the injured renal tubules in UUO kidneys (**C**).

Interestingly, these tdTomato positive cells and the other TNC reporter eGFP positive cells in the UUO model were predominantly located adjacent to the tubular structures that were dilated and negative for LTL, THP (Tamm-Horsfall protein, a marker of the thick ascending limb), AQP2, and CD31 (Fig. 7A–C and Supplementary Fig. 4). To examine whether these tubular structures surrounded by TNC reporter positive cells were injured renal tubules, we co-labeled TNC reporter with LTL or antibodies against THP or AQP2 on serial sections. This serial section study showed that the tubules surrounded by TNC-expressing cells had continuation to the tubules with positive AQP2 staining, supporting that TNC-expressing cells were localized surrounding the injured tubular epithelial cells in UUO kidneys (Fig. 7C and Supplementary Fig. 4C).

### TNC-expressing cells in fibrotic kidneys were mostly PDGFRβ^+^NG2^+^ fibroblasts

**Fig. 8:**
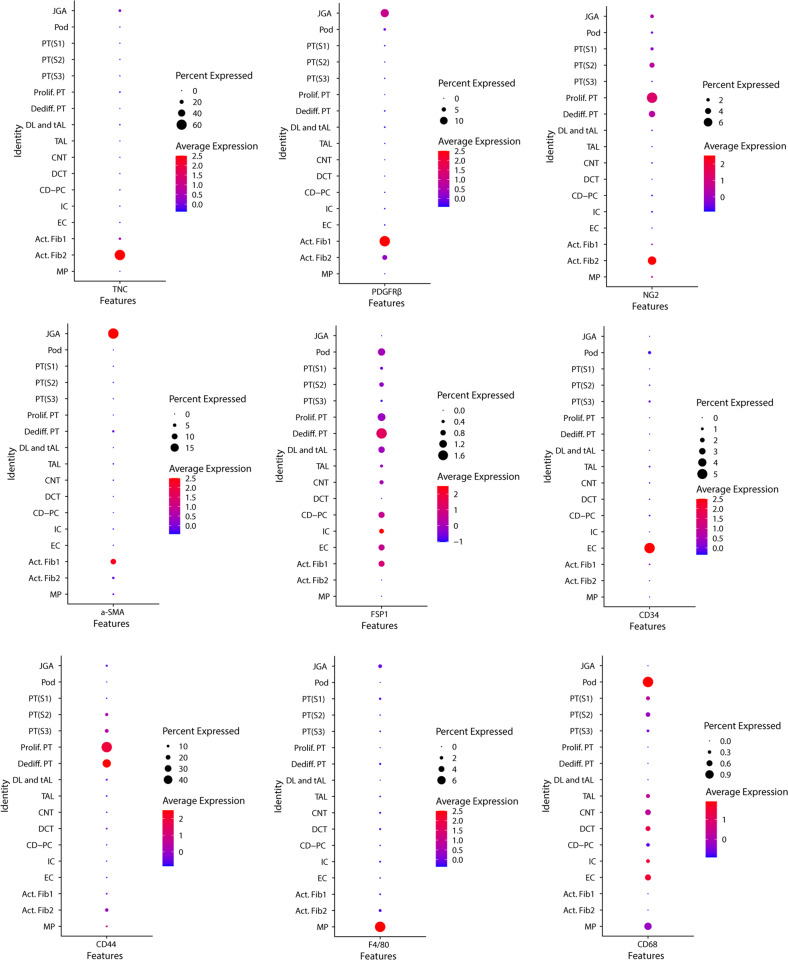
Single nucleus RNA-seq identified TNC-expressing fibroblasts in mouse UUO kidneys. A previously generated single nucleus RNA-seq dataset profiling kidney of mouse UUO model at day 14 was used for re-analysis. Two subpopulations of activated fibroblasts were identified, with distinct TNC-expression pattern. Around 60% of activated fibroblast 2 (Act.Fib2) expressed TNC, while less than 5% of activated fibroblast 1 expressed TNC. It was observed that around 6% of activated fibroblast 2 expressed PDGFRβ, NG2 and CD44. Fibroblasts were almost completely negative in FSP1, CD34, F4/80 and CD68. JGA juxtaglomerular apparatus; Pod podocyte; PT proximal tubule; Prolif proliferating; Dediff dedifferenciated; DL descending limb; tAL thin ascending limb; TAL thick ascending limb; CNT connecting tubule; DCT distal convoluted tubule; CD-PC collecting duct-principal cell; IC intercalated cell; EC endothelial cell; Act activating; Fib fibroblast; MP macrophage.

To investigate TNC expression in the fibrotic kidneys, we used a previously generated single nucleus RNA-seq dataset profiling kidney of mouse UUO model at day 14 (Supplementary Fig. 5A–C). Two subpopulations of activated fibroblasts were identified, with distinct TNC-expression pattern. Around 60% of activated fibroblast 2 (Act.Fib2) expressed TNC, while less than 5% of activated fibroblast 1 expressed TNC. Six percent of the activated fibroblast 2 expressed PDGFRβ, NG2 and CD44. Fibroblasts were almost completely negative for FSP1, CD34, F4/80 and CD68 (Fig. 8).

**Fig. 9:**
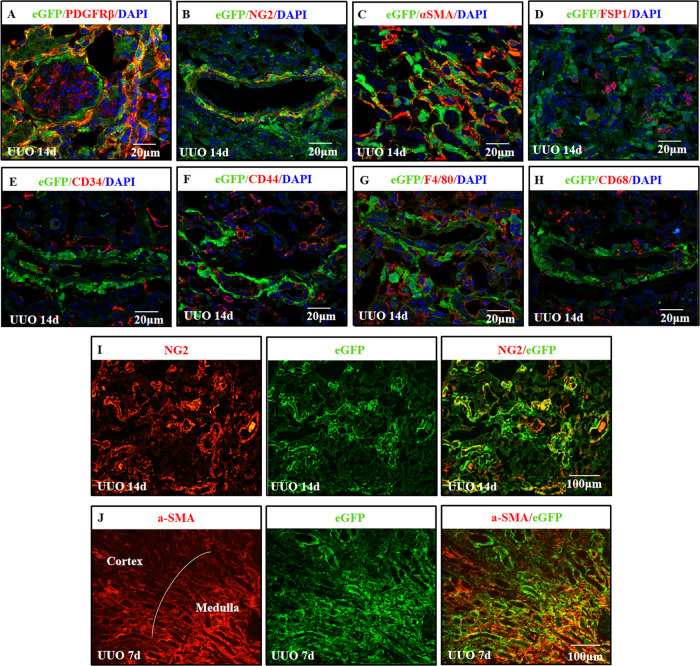
TNC-expressing cells in fibrotic kidneys were mostly PDGFRβ + NG2 + fibroblasts. In TNC^CreER2-eGFP/+^ reporter mice, eGFP positive cells were all PDGFRβ positive, consistent with stromal cells (**A**). Most of the eGFP-positive cells were also NG2 (a marker of pericyte) positive (**B**, **I**). Some of eGFP positive cells were αSMA positive, while most of αSMA positive myofibroblasts were not eGFP positive (**C**, **J**). These eGFP-positive cells were not positive for FSP1 (**D**). They were neither CD34 nor CD44 positive progenitors (**E**, **F**), and were neither CD68 nor F4/80 positive (**G**, **H**). (*n* = 4, 3 sections for each).

To characterize the cells that express TNC in the fibrotic kidneys, we co-labeled TNC reporter eGFP with specific markers in UUO model. We did not use tdTomato as reporter because its autofluorescence is too strong that it can be detected in the green channel. We found that the eGFP-positive cells were positive for PDGFRβ, consistent with stromal cells (Fig. 9A and Supplementary Fig. 6A). Most of eGFP-positive cells were NG2 (a marker of pericyte) positive (Fig. 9B, I and Supplement Fig. 6B). Some of the eGFP-positive cells were αSMA positive, while most of αSMA positive myofibroblasts were not eGFP positive (Fig. 9C, J and Supplement Fig. 6C). None of the eGFP positive cells were positive for FSP1 (Fig. 9D and Supplementary Fig. 6D). In addition, the eGFP positive cells were neither CD34 nor CD44 positive (Fig. 9E, F and Supplementary Fig. 6E, F), and were neither CD68 nor F4/80 (markers of macrophages) positive (Fig. 9G, H and Supplementary Fig. 6G, H).

In addition, it has been reported that renal papilla contains stem cells [34], and TNC has also been reported to be expressed in stem cells [35]. To determine whether the TNC-expressing cells in normal renal medulla were progenitors or source of the expanded stromal cells in the renal cortex after fibrosis, we conducted a cell linage tracing study in TNC^CreER2-eGFP/+^;R26^tdTomato/+^ mice. We found that the expanded TNC-expressing cells in the fibrotic renal cortex were not originated from the constitutive TNC-expressing cells in the renal papilla following UUO (Supplementary Fig. 7).

## Discussion

Fibrosis is a common event that leads to irreversible loss of organ function and organ failure, and so far has no effective therapy [36]. The excessive deposition of ECM is a key feature of fibrosis. Accumulating evidence has revealed that ECM is not only the product of fibrosis, but also actively drives the persistence of fibrosis [3, 4, 6]. TNC is an ECM glycoprotein that is under tight temporal and spatial regulation [37, 38]. A recent study suggested that TNC played an important role in kidney fibrosis [18]. Our study showed that (1) the TNC-expression levels and the number of TNC-expressing cells dramatically increased during fibrosis in the kidney; (2) deletion of TNC attenuated kidney fibrosis; (3) TNC facilitated fibrosis, at least in part, by promoting the phosphorylation of STAT3; (4) the cells expressing TNC were mainly localized surrounding the injured tubules, suggesting stromal cells in response to epithelium damage; (5) the cells that produced TNC were a special population of stromal cells, most of which were positive for NG2 and only a few positive for αSMA.

TNC was significantly increased in the kidney with fibrosis induced by UUO or IR injury. Since TNC deficiency significantly increased the severity of acute kidney injury after IR [39], which made the association between TNC and fibrosis more complicated, we investigated the role of TNC in fibrosis and explored the mechanism using UUO model. Our study provided strong evidence that TNC played an important role in promoting kidney fibrosis using a TNC knockout mouse line, consistent with a previous study using siRNA-TNC injection approach [18]. In chronic kidney disease progression, tubular epithelia have been reported to sense and response to damage [40–43], followed by interstitial stroma activation [44]. Our study showed that the TNC-expressing cells (eGFP positive cells) in the fibrotic cortex were primarily localized around the injured tubules that had lost their epithelial markers. This specific localization may indicate that TNC plays an important role in linking tubule injury and interstitial cell activation and fibrosis. Interestingly, TNC was reported to be expressed only in the mesenchyme surrounding the epithelia undergoing differentiation during embryonic development [45], and was induced by epithelial-mesenchymal interactions where epithelia were undergoing differentiation or restoration after injury [46]. TGFβ, a well-established driver of fibrosis, was found to be upregulated in the injured tubular epithelium [47, 48]. Our study showed that TNC was, at least partially, induced by TGFβ (Supplementary Fig. 8). Taken together, these data highlight a potential role of TNC in linking tubule damage to the persistence of interstitial fibrosis.

TNC is a matricellular protein with multiple domains including EGF-like repeats, FNIII repeats and fibrinogen-like globe [13]. These domains can serve as ligands of cell-surface receptors and activate intracellular signals. Receptors for TNC include EGFR, integrins and toll-like receptors [49–52]. These receptors have an enzymatic activity in intracellular domain and usually regulate downstream kinase cascade. Therefore, we screened the kinases and identified STAT3 as a potential signaling pathway mediating the effect of TNC. In UUO-induced kidney fibrosis models, deficiency of TNC gene significantly reduced the phosphorylation of STAT3. In cultured cells, TNC promoted fibroblast proliferation by upregulating the phosphorylation of STAT3, because STAT3 inhibitor blocked the cells proliferation induced by TNC. STAT3 is a transcription factor and has been well demonstrated as a downstream target of EGFR [53, 54]. Although we also found that EGFR inhibition could block the role of TNC on cell proliferation, we did not find that TNC bound with EGFR by co-immunoprecipitation, which may be because the affinity between EGF-like repeats and EGFR is low [49]. Mounting evidence has indicated that STAT3 activation is associated with the proliferation of various cells including fibroblasts and the development of fibrosis [55–58]. It has been demonstrated that STAT3 promotes cell cycle progression and proliferation by inducing the expression of the target genes, such as cyclin D1 and MYC [59, 60]. STAT3 and its substrates are overexpressed in a wide range of hematopoietic malignancies and solid tumors, and contribute to the proliferative drive [61].

A single nucleus RNA-seq dataset profiling kidney of mouse UUO model at day 14 showed that TNC was predominantly expressed in Act.Fib2 but not Act.Fib1 [20]. Using this dataset, we found that around 60% of Act.Fib2 expressed TNC while less than 5% of Act.Fib1 expressed TNC, and around 6% Act.Fib2 also expressed NG2, PDGFRβ and CD44. NG2 is a transmembrane proteoglycan and usually used to label pericytes (NG2^+^PDGFRβ^+^) which had been suggested to be a precursor of myofibroblasts [62]. NG2 expression has also been identified in several types of immature cells (such as progenitor and tumor cells) and is downregulated when cells become mature and quiescent [32, 63, 64]. Similarly, TNC expression has also been found in developing cells, such as progenitors during development, tumor stroma and mesenchymal stem cells [35, 46, 65]. Since co-expression analysis in snRNA-seq might have greatly underestimated the percentage of co-expressing cells due to dropout problem, we co-stained TNC reporter eGFP with these markers and confirmed that most of the TNC-expressing cells were positive for both NG2 and PDGFRβ, but not for CD44 (CD 44 is mainly expressed by dedifferentiated or proliferating proximal tubules). Myofibroblasts, characterized by positive staining for αSMA, are the key contributor of kidney fibrosis [66]. It is well known that myofibroblasts are the major source of ECM [67]. Our study showed only some of eGFP-positive cells were αSMA positive, while most of αSMA positive myofibroblasts were not eGFP positive. This result was consistent with the single nucleus RNA-seq analysis, in which αSMA was more expressed by Act.Fib1 rather than Act.Fib2.

It has been reported that renal papilla contains stem cells or progenitors which repopulate after kidney injury [34]. TNC is constitutively expressed in the renal medullary interstitial cells [19]. To examine whether these TNC-expressing cells in renal medulla were progenitors that migrated to renal cortex after injury, we used a genetic mouse model that specifically tracked and fate-mapped renal medullary interstitial TNC-expressing cells. The results demonstrated that the TNC-expressing fibroblasts in renal cortex during fibrosis were not originated from papilla. The origin of TNC-expressing fibroblasts after injury remains unclear and needs further studies.

## Conclusions

In conclusion, our study provides strong evidence that non-structural matrix protein TNC that contains multiple functional domains plays an important role in kidney fibrosis. TNC is mostly expressed by NG2^+^PDGFRβ^+^ cells around the injured tubules and STAT3 is a signaling pathway at least partially mediating the profibrotic effect of TNC. TNC pathway may serve as a potential therapeutic target to treat interstitial fibrosis and the progression of kidney injury.

## Supplementary information

Supplement Figure 1-1

Supplement Figure 1-2

Supplement Figure 2

Supplement Figure 3

Supplement Figure 4

Supplement Figure 5

Supplement Figure 6-1

Supplement Figure 6-2

Supplement Figure 7

Supplement Figure 8

Original Data File

Reproducibility checklist

## Data Availability

Data sharing not applicable to this article as no datasets were generated or analyzed during the current study.
