# Supplementary figures and images for: Matrix protein Tenascin-C promotes kidney fibrosis via STAT3 activation in response to tubular injury
Source: Cell Death Dis. 2022 Dec 15;13(12):1044. doi: 10.1038/s41419-022-05496-z (PMC9755308; doi:10.1038/s41419-022-05496-z)

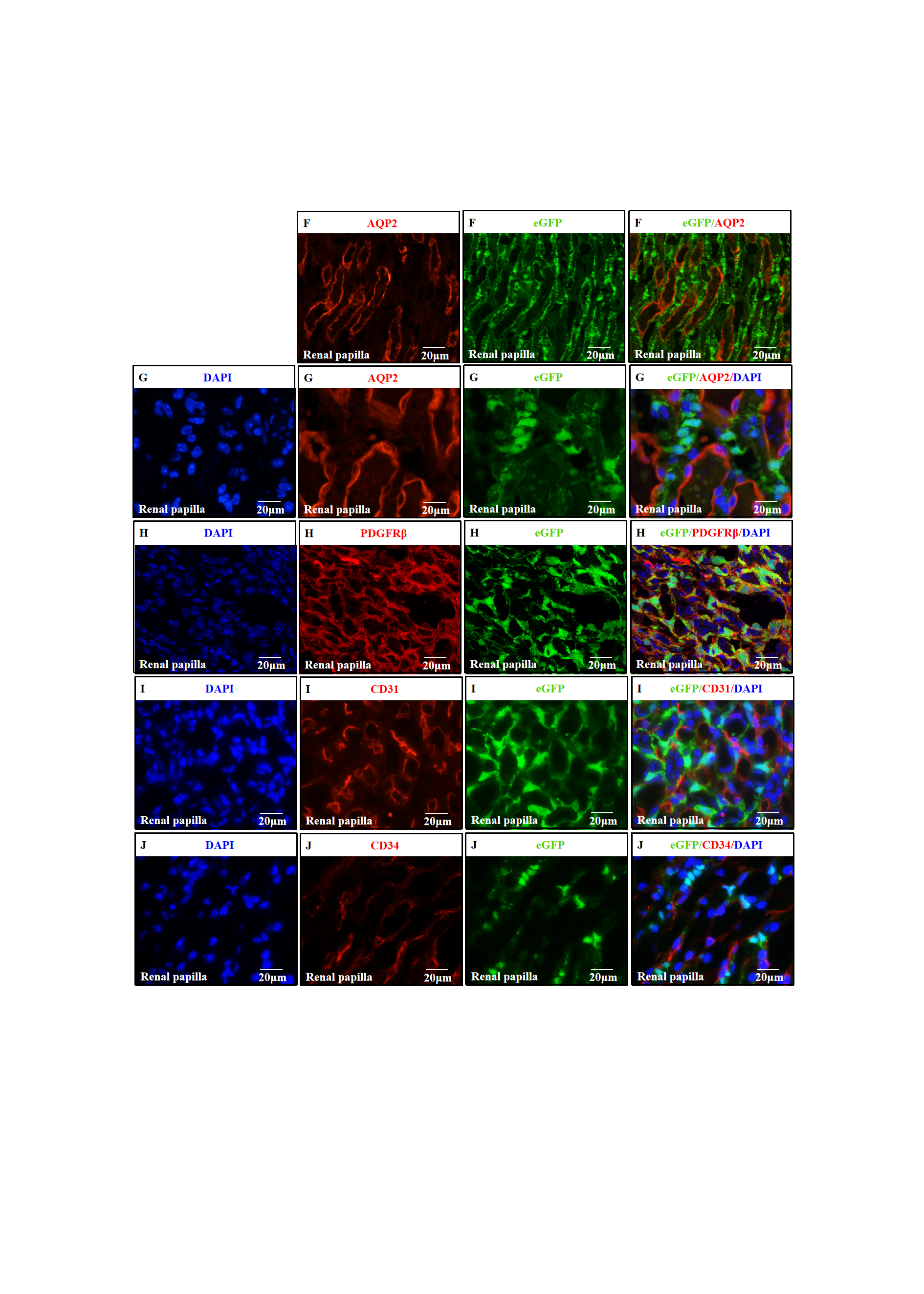

Supplement: Supplementary file 1 — Supplement Figure 1-1 [file 41419_2022_5496_MOESM1_ESM.tif]

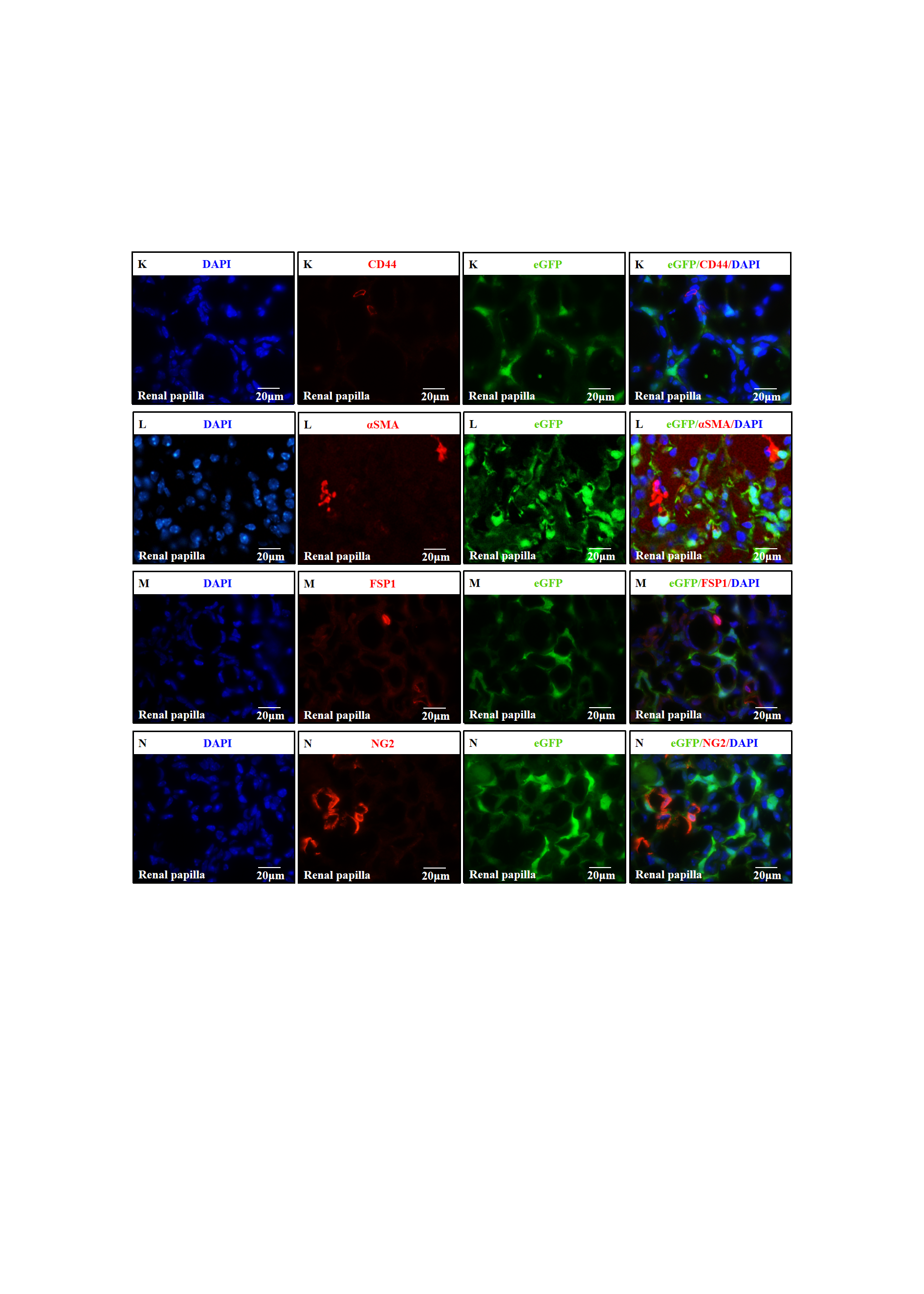

Supplement: Supplementary file 2 — Supplement Figure 1-2 [file 41419_2022_5496_MOESM2_ESM.tif]

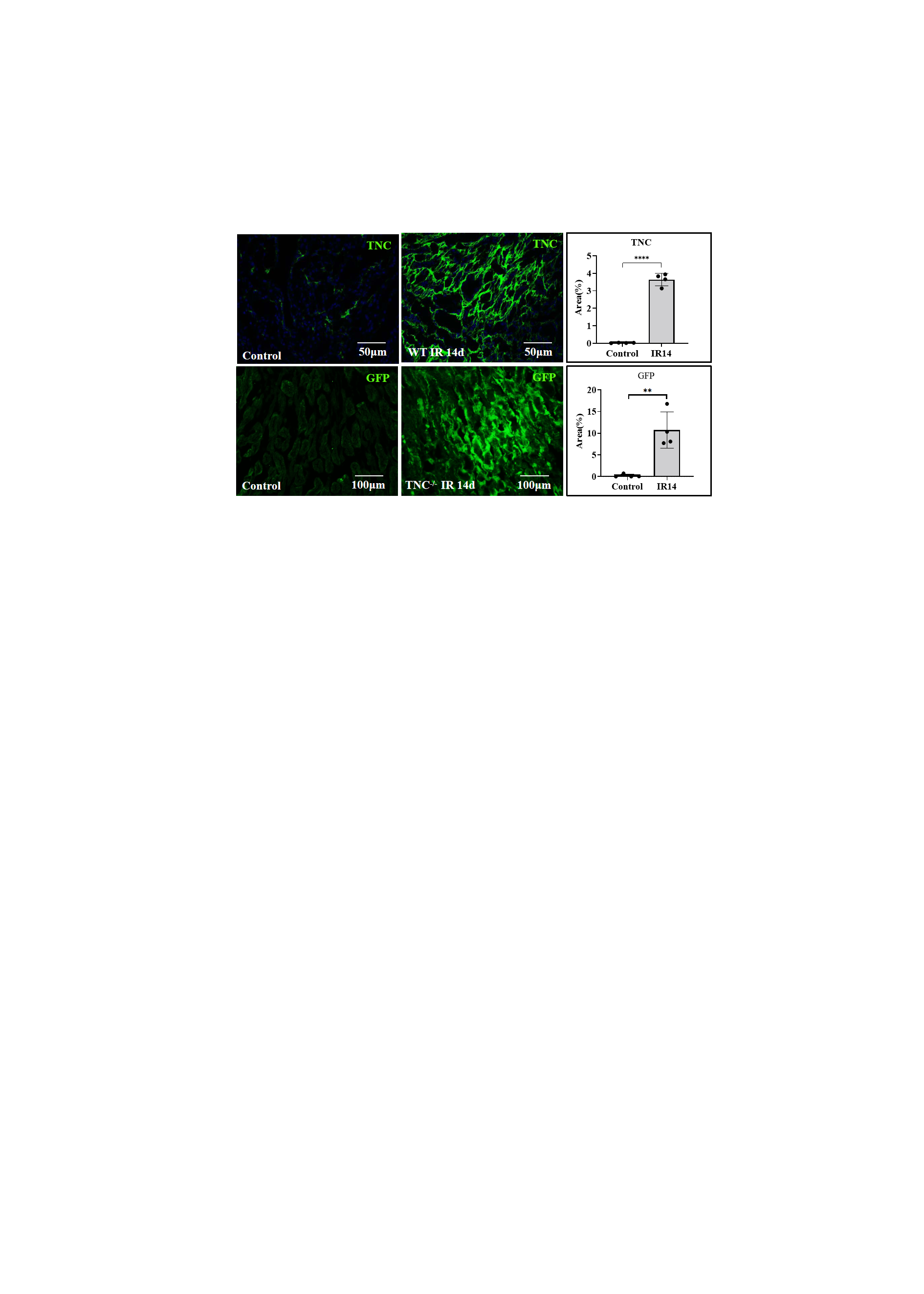

Supplement: Supplementary file 3 — Supplement Figure 2 [file 41419_2022_5496_MOESM3_ESM.tif]

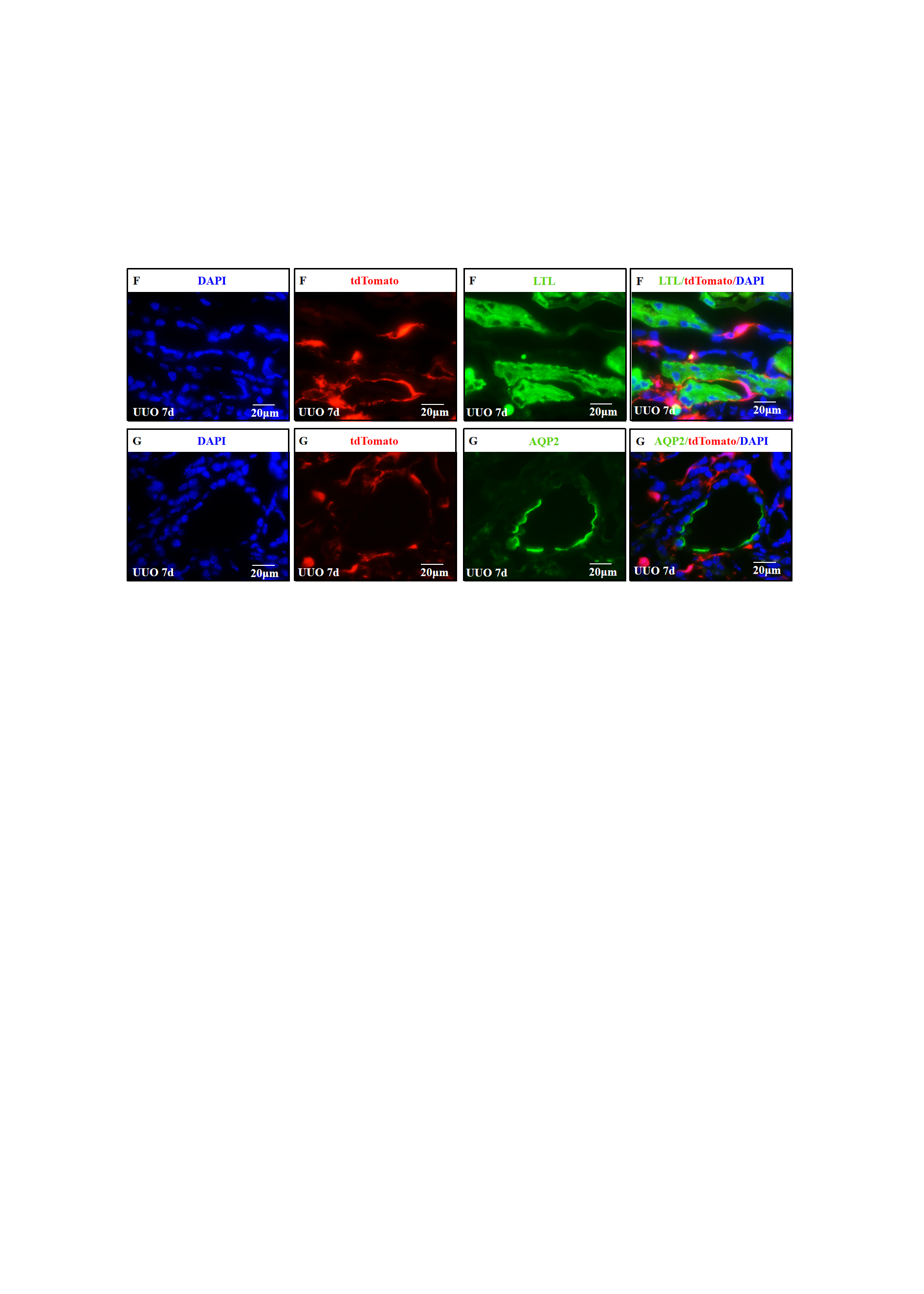

Supplement: Supplementary file 4 — Supplement Figure 3 [file 41419_2022_5496_MOESM4_ESM.tif]

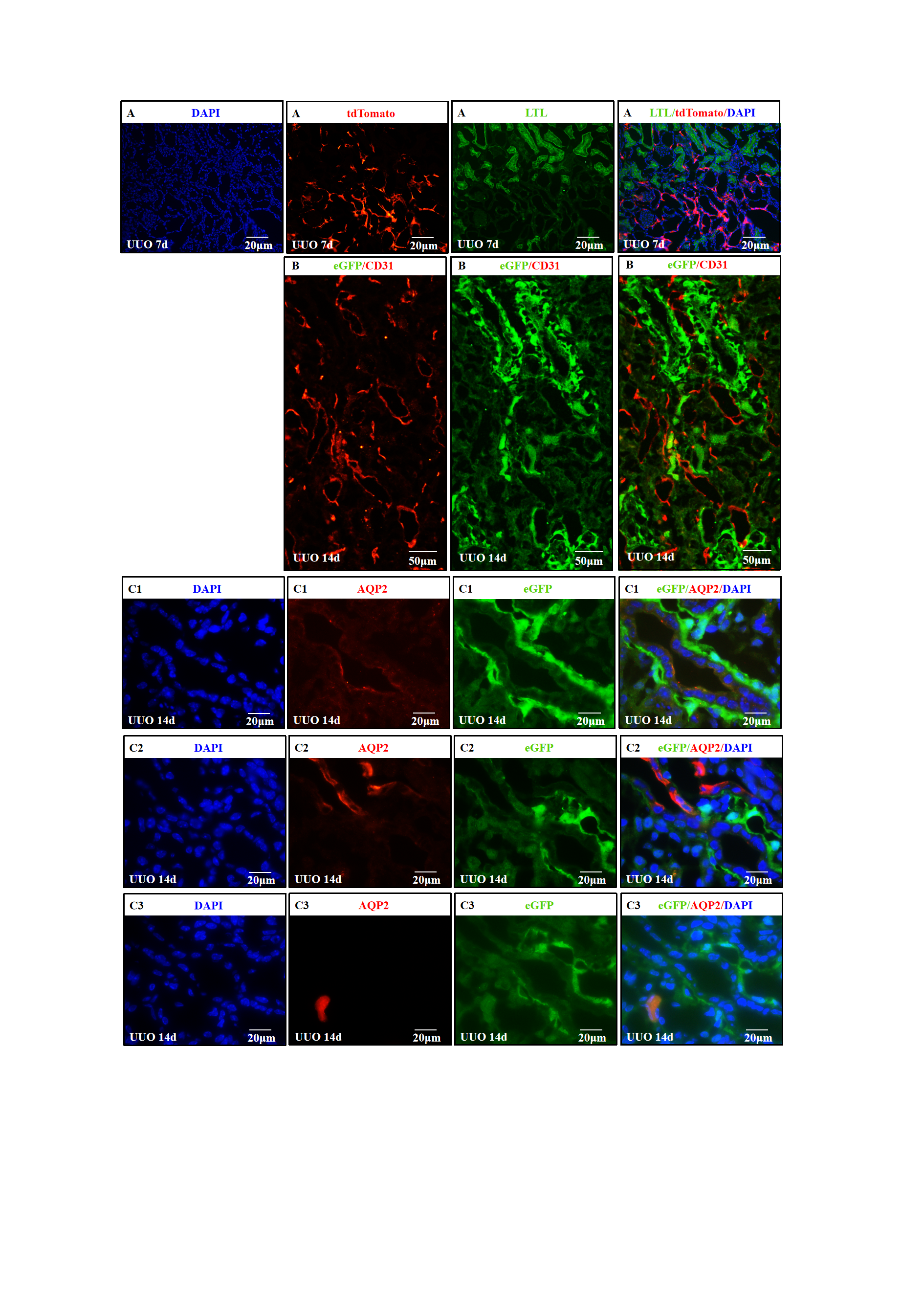

Supplement: Supplementary file 5 — Supplement Figure 4 [file 41419_2022_5496_MOESM5_ESM.tif]

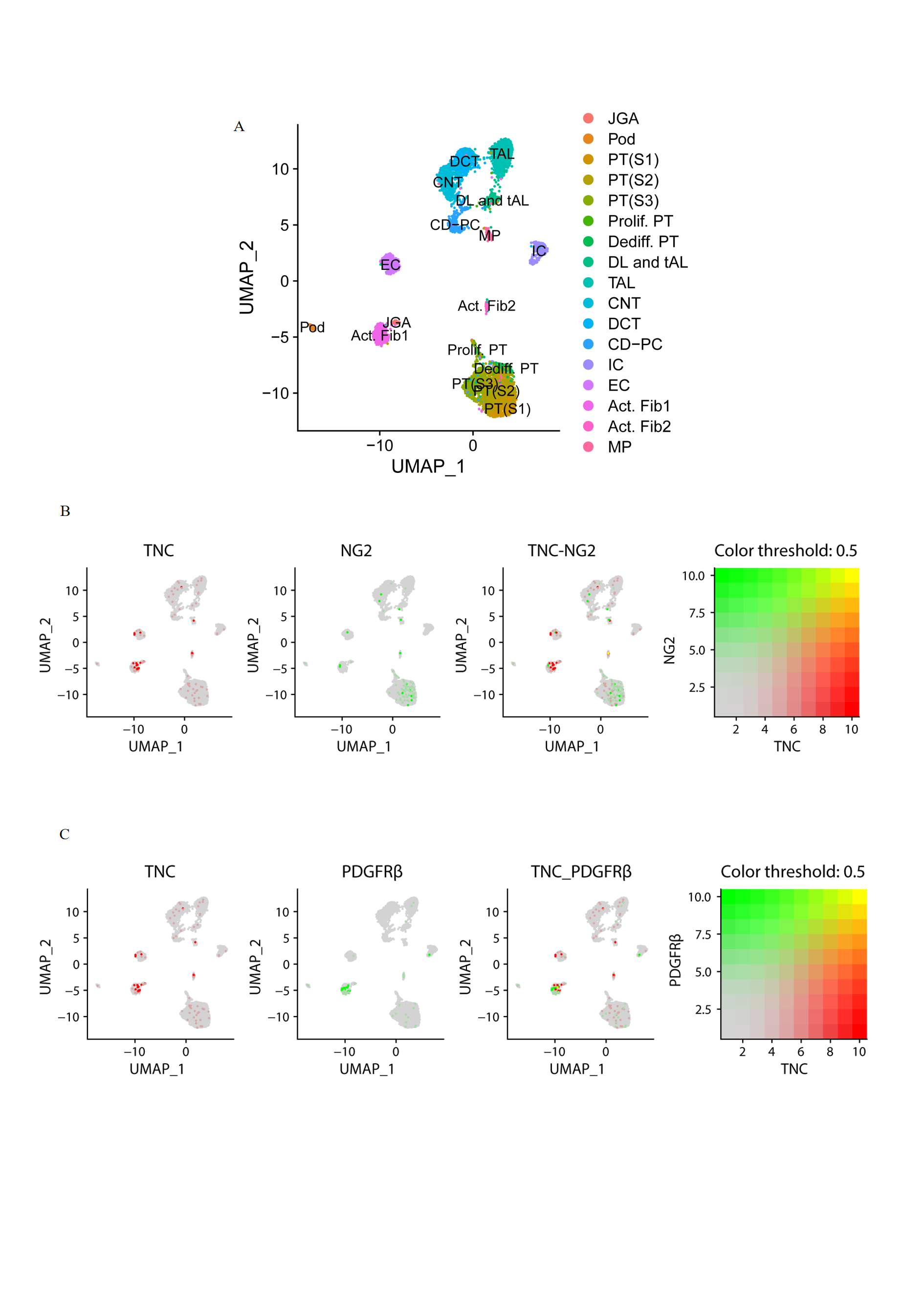

Supplement: Supplementary file 6 — Supplement Figure 5 [file 41419_2022_5496_MOESM6_ESM.tif]

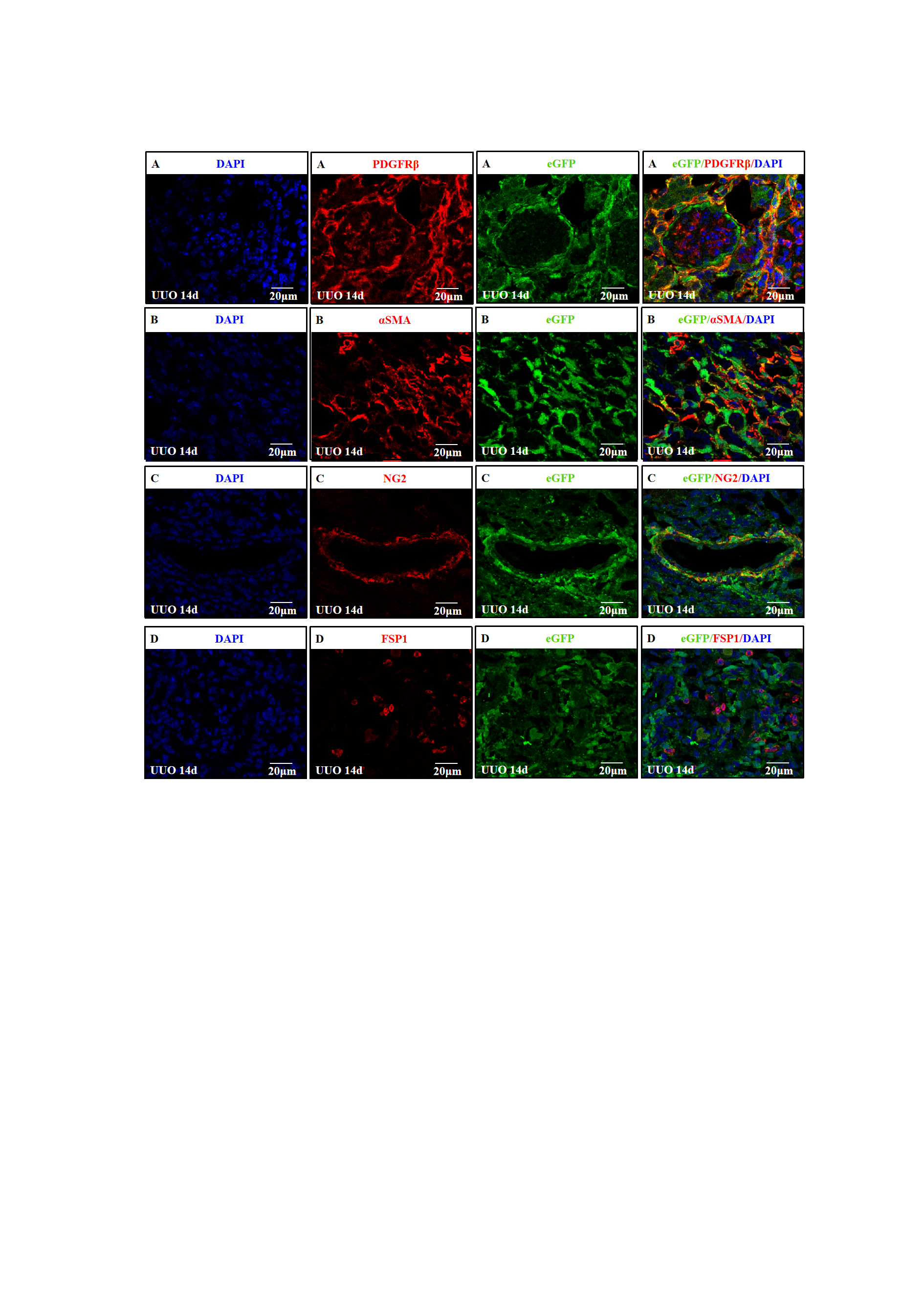

Supplement: Supplementary file 7 — Supplement Figure 6-1 [file 41419_2022_5496_MOESM7_ESM.tif]

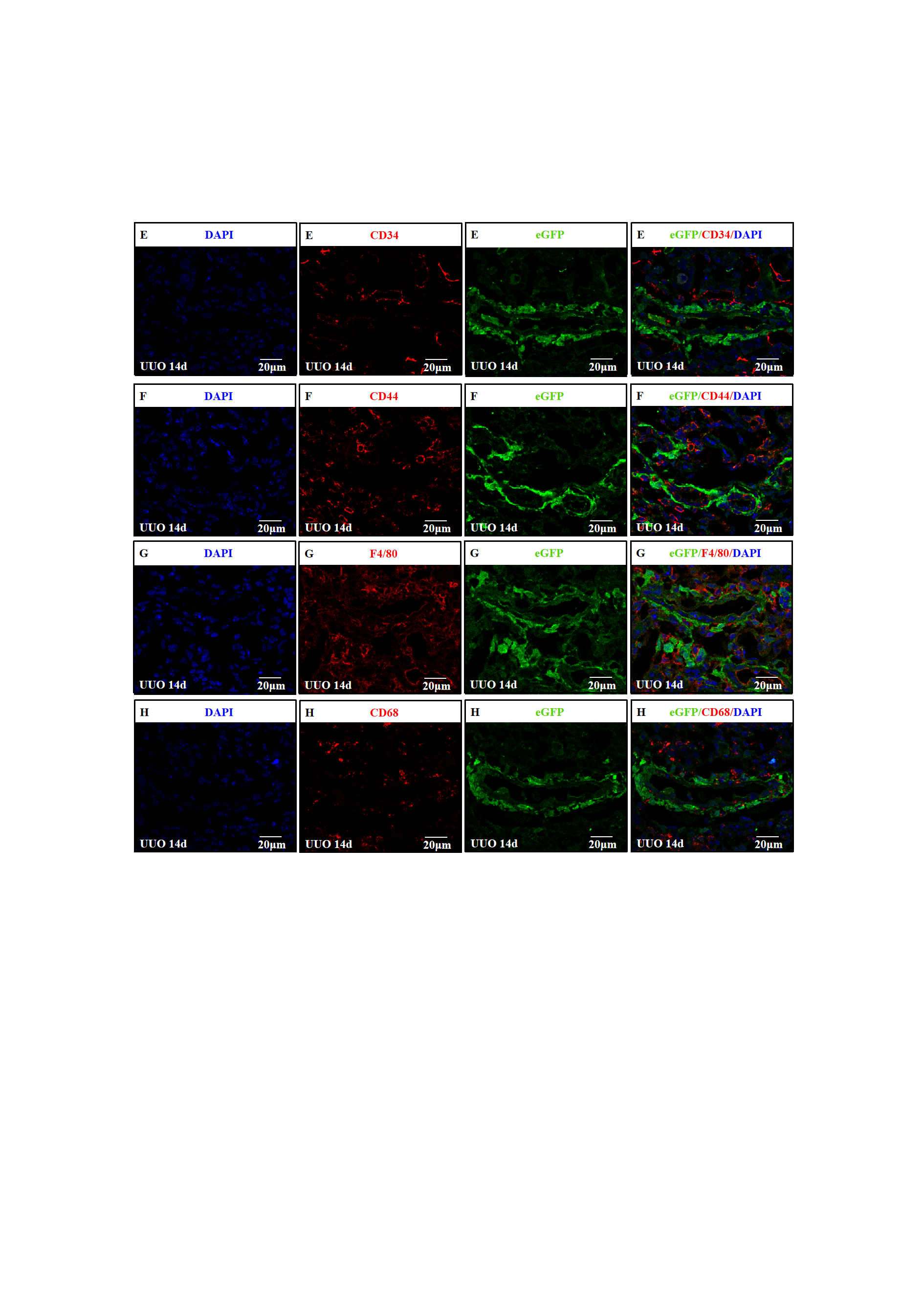

Supplement: Supplementary file 8 — Supplement Figure 6-2 [file 41419_2022_5496_MOESM8_ESM.tif]

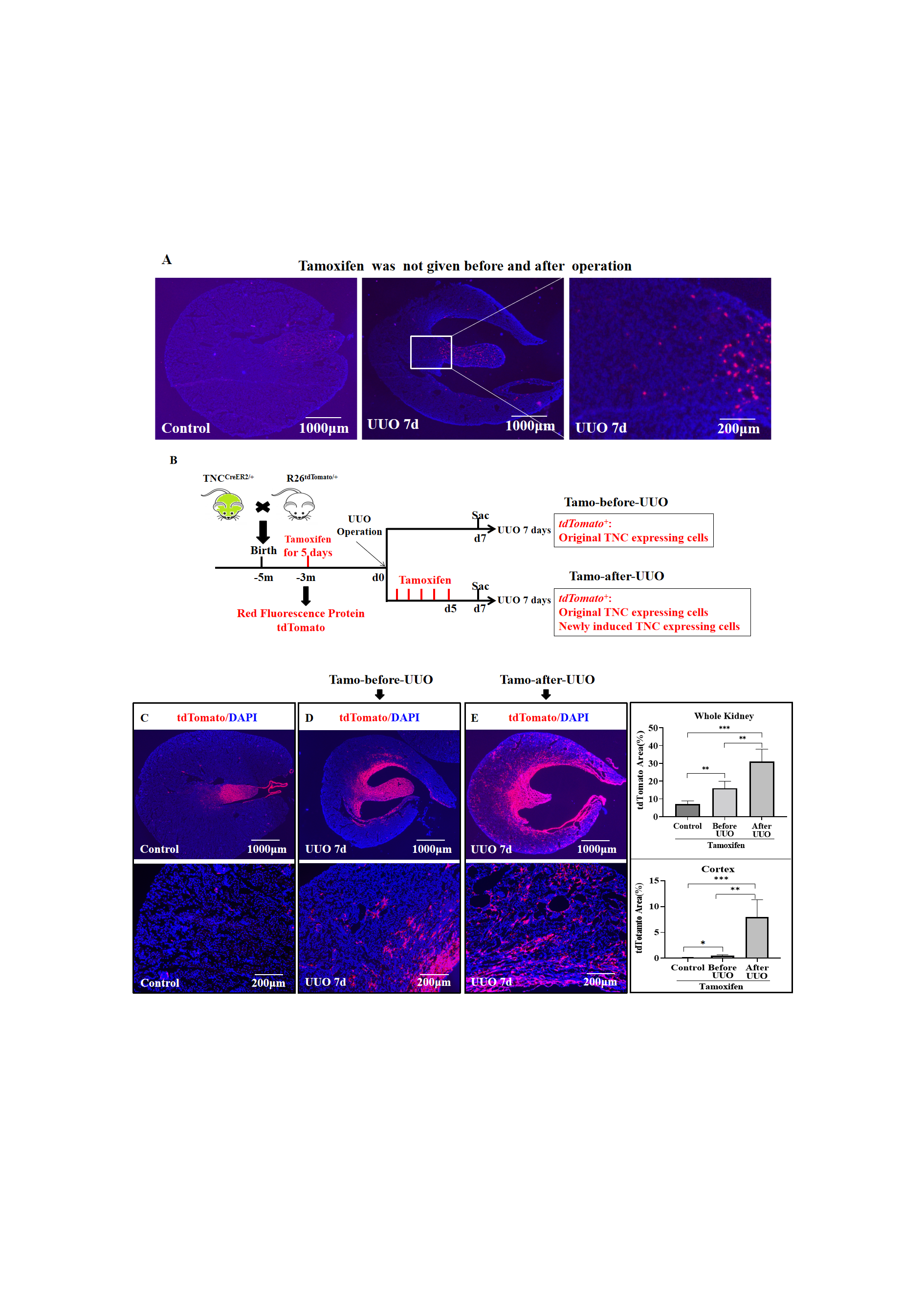

Supplement: Supplementary file 9 — Supplement Figure 7 [file 41419_2022_5496_MOESM9_ESM.tif]

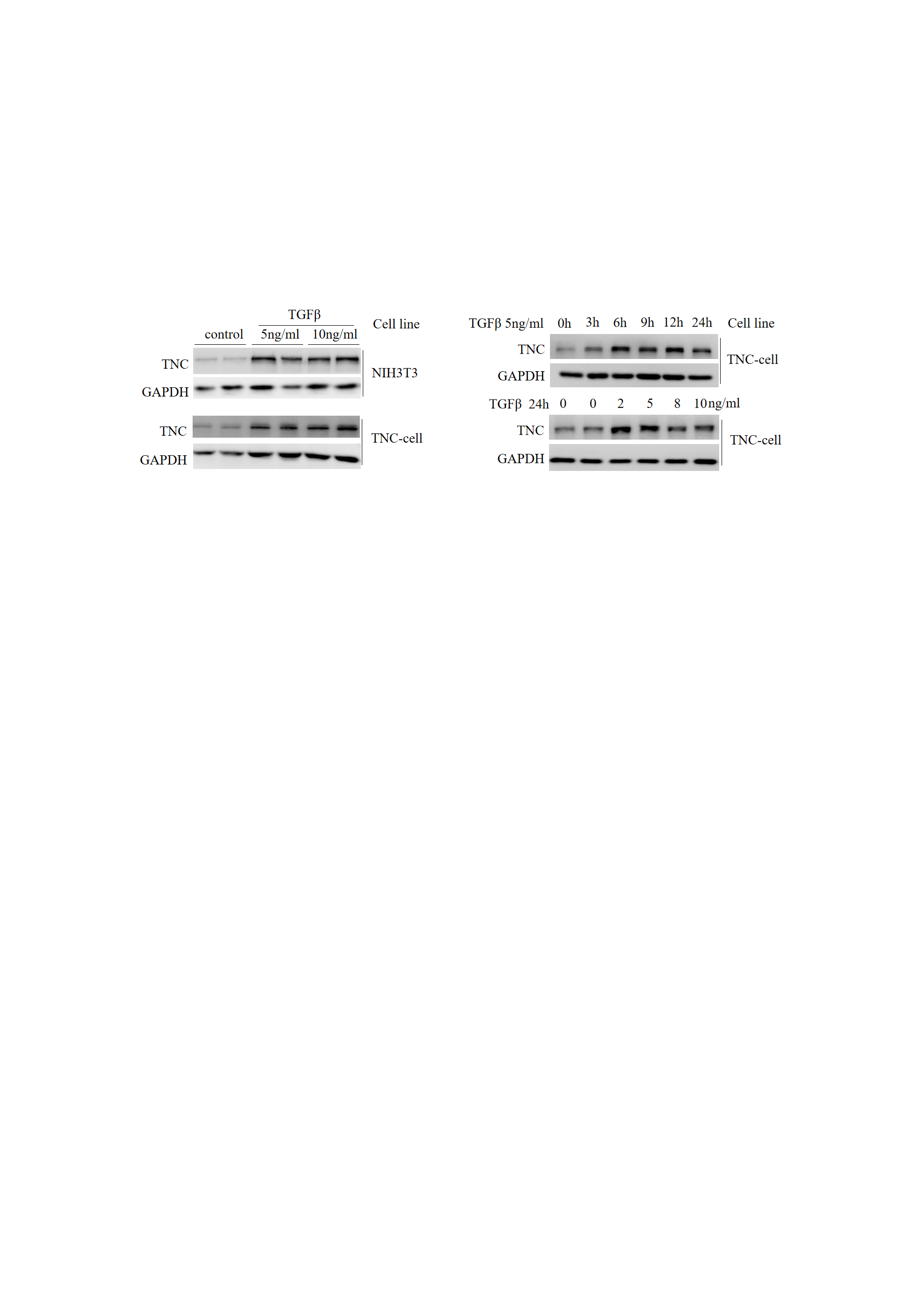

Supplement: Supplementary file 10 — Supplement Figure 8 [file 41419_2022_5496_MOESM10_ESM.tif]
