# Supplementary material for: Matrix protein Tenascin-C promotes kidney fibrosis via STAT3 activation in response to tubular injury
Source: Cell Death Dis. 2022 Dec 15;13(12):1044. doi: 10.1038/s41419-022-05496-z (PMC9755308; doi:10.1038/s41419-022-05496-z)

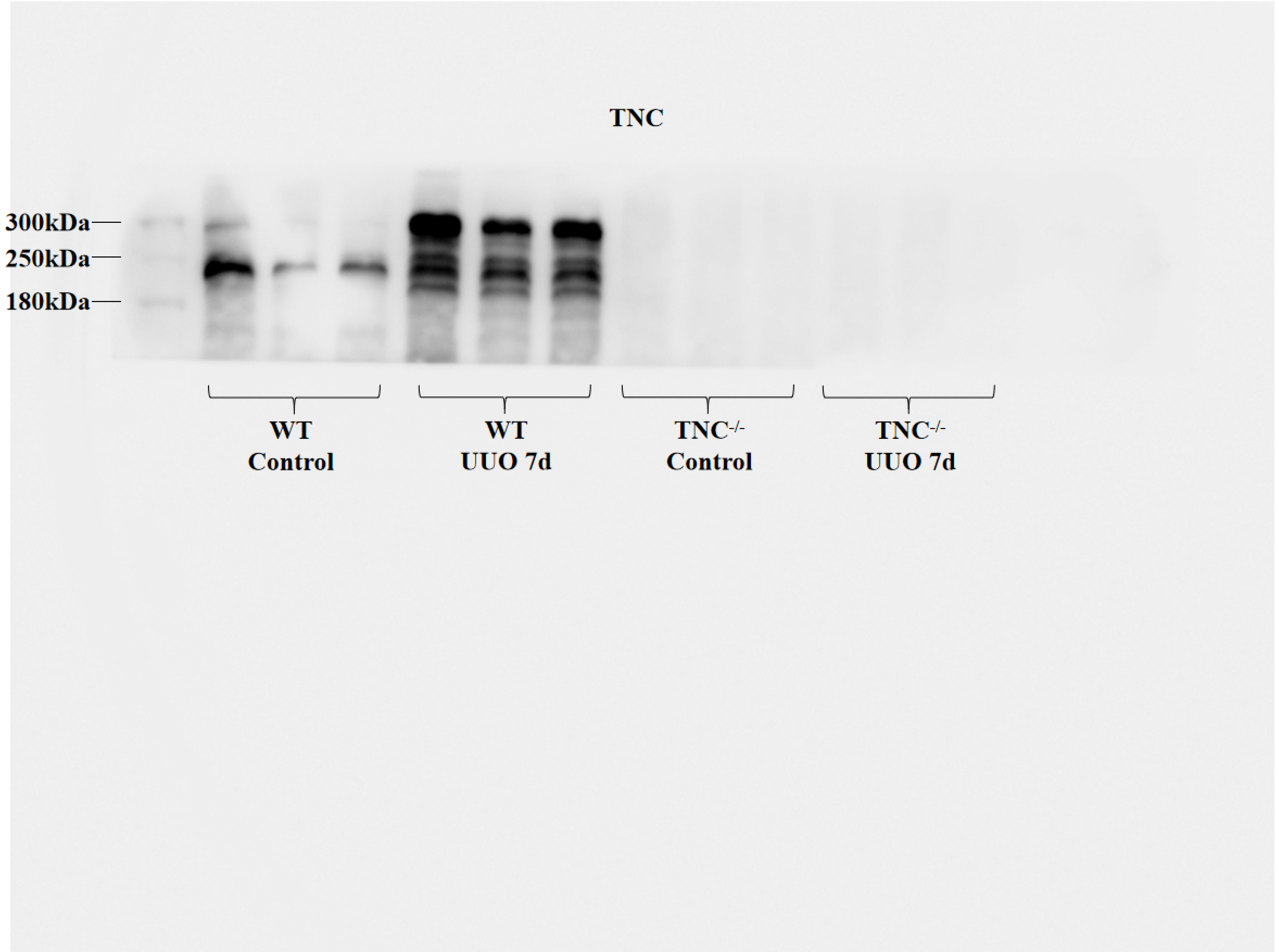
TNC
300kDa
250kDa
180kDa
WT
Control
WT
UUO 7d
TNC-/-
Control
TNC-/-
UUO 7d

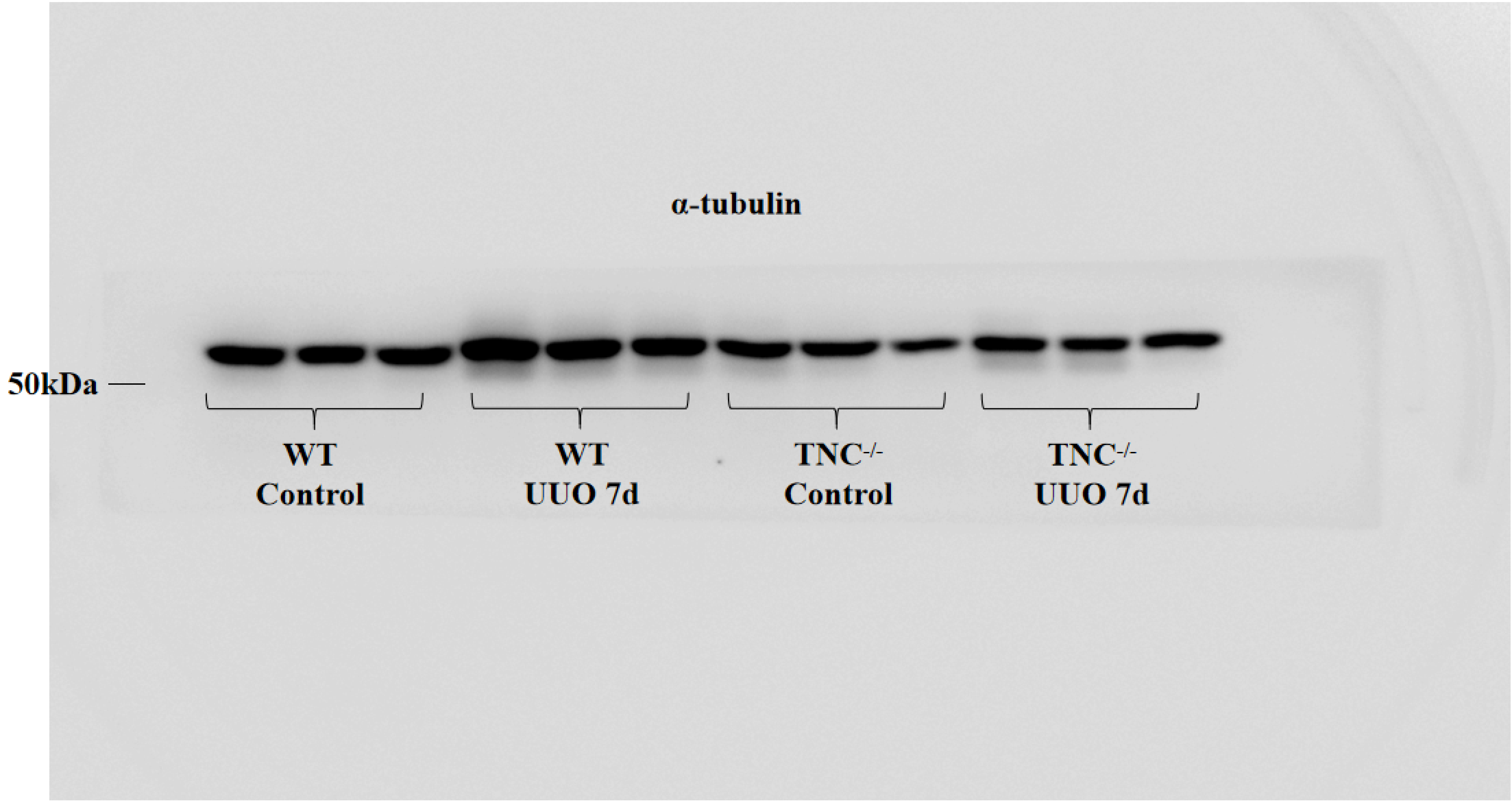
α-tubulin
50kDa
WT
Control
WT
UUO 7d
TNC-/-
Control
TNC-/-
UUO 7d

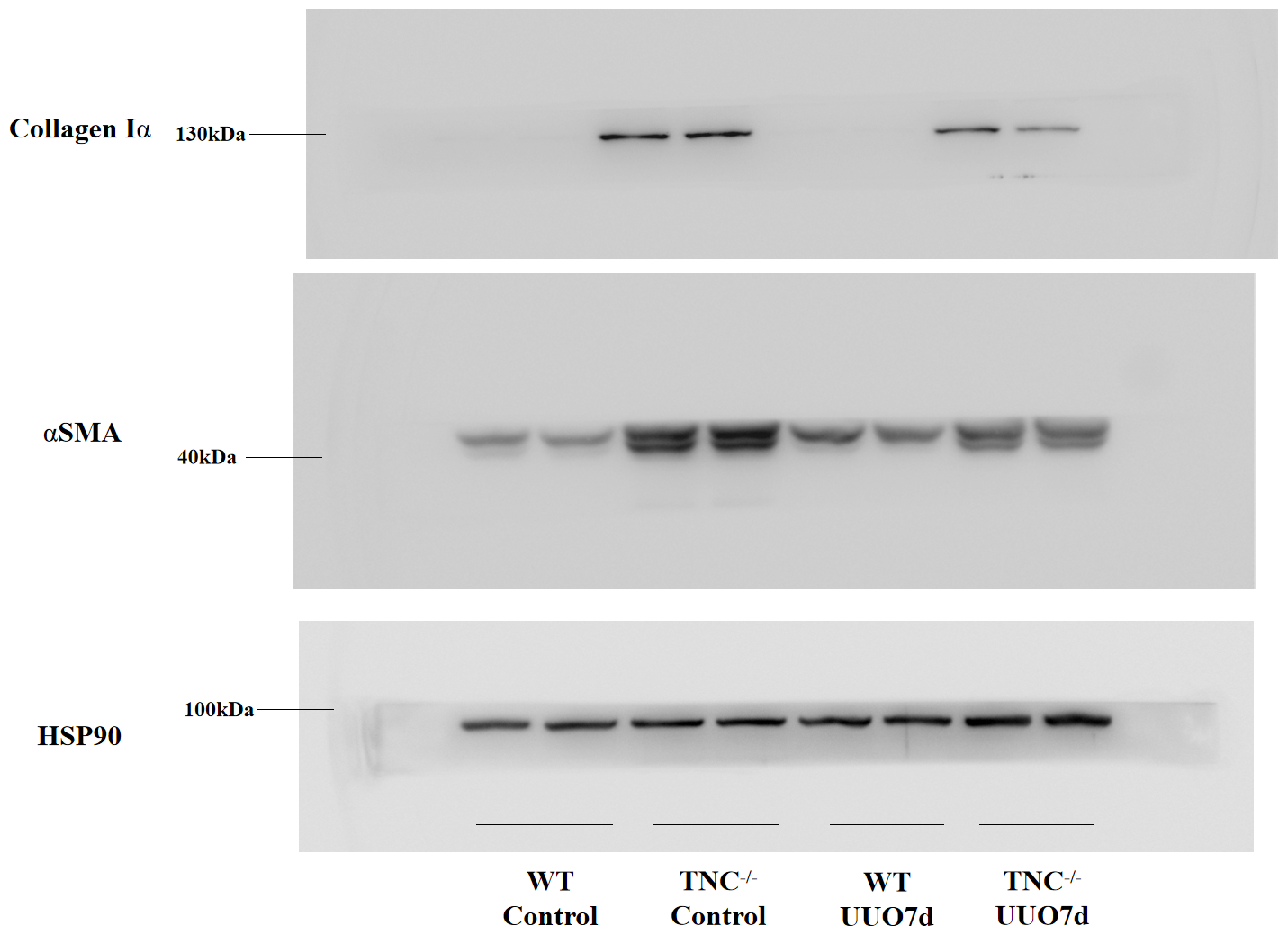
Collagen Iα
130kDa
αSMA
40kDa
100kDa
HSP90
WT Control
TNC-/- Control
WT UUO7d
TNC-/- UUO7d

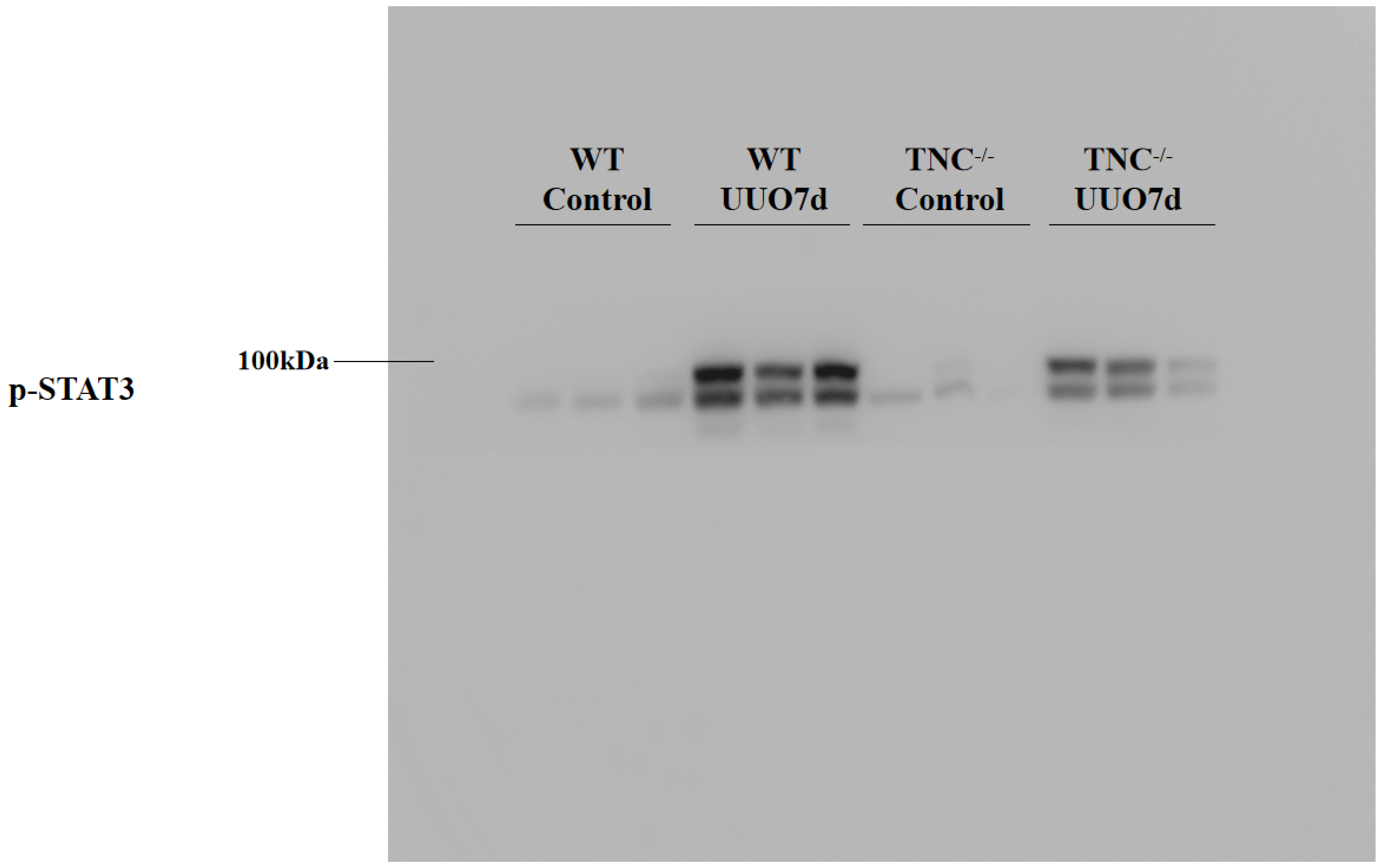
WT Control
WT UUO7d
TNC-/- Control
TNC-/- UUO7d
p-STAT3
100kDa

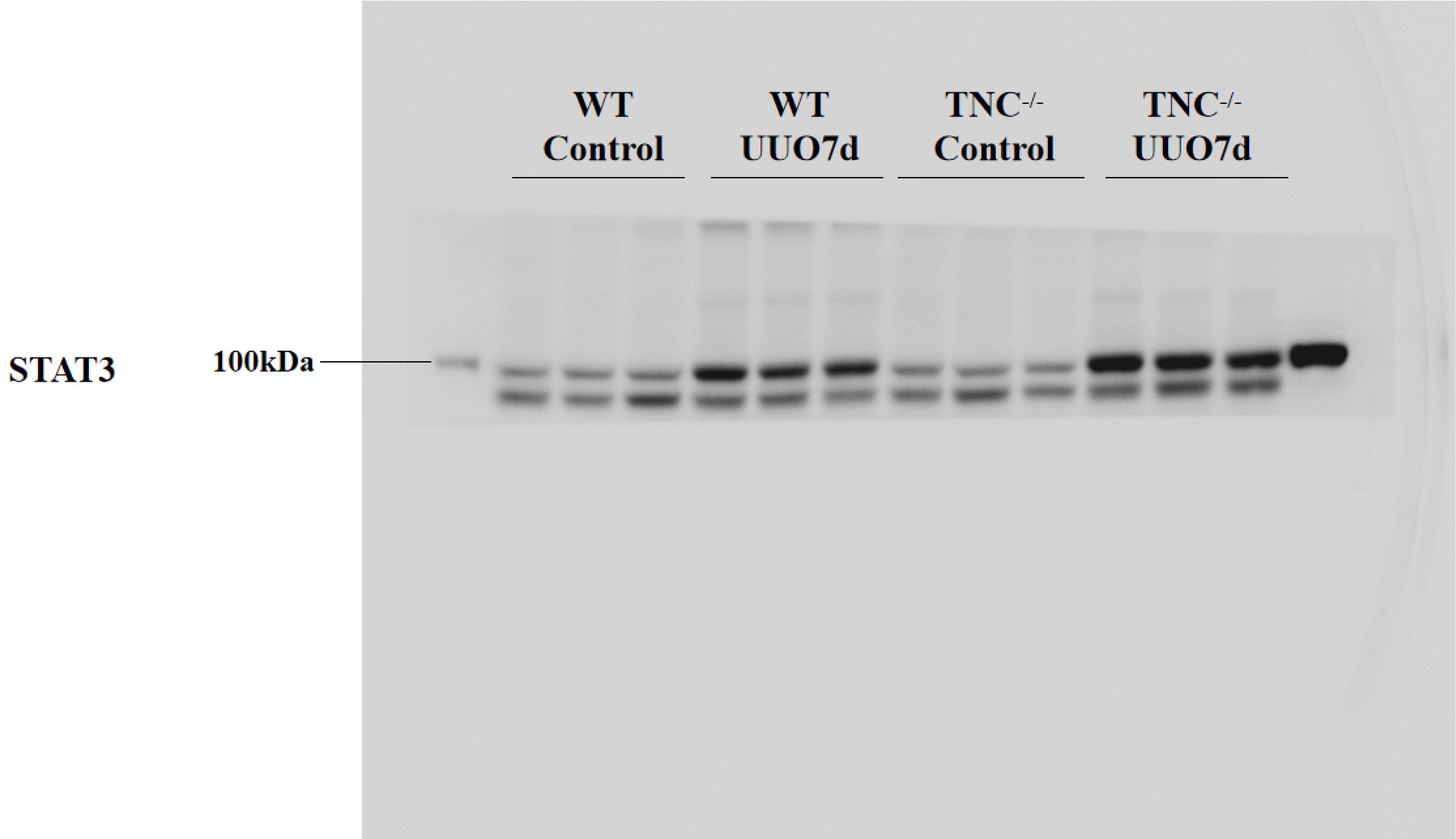
WT Control
WT UUO7d
TNC-/- Control
TNC-/- UUO7d
STAT3
100kDa

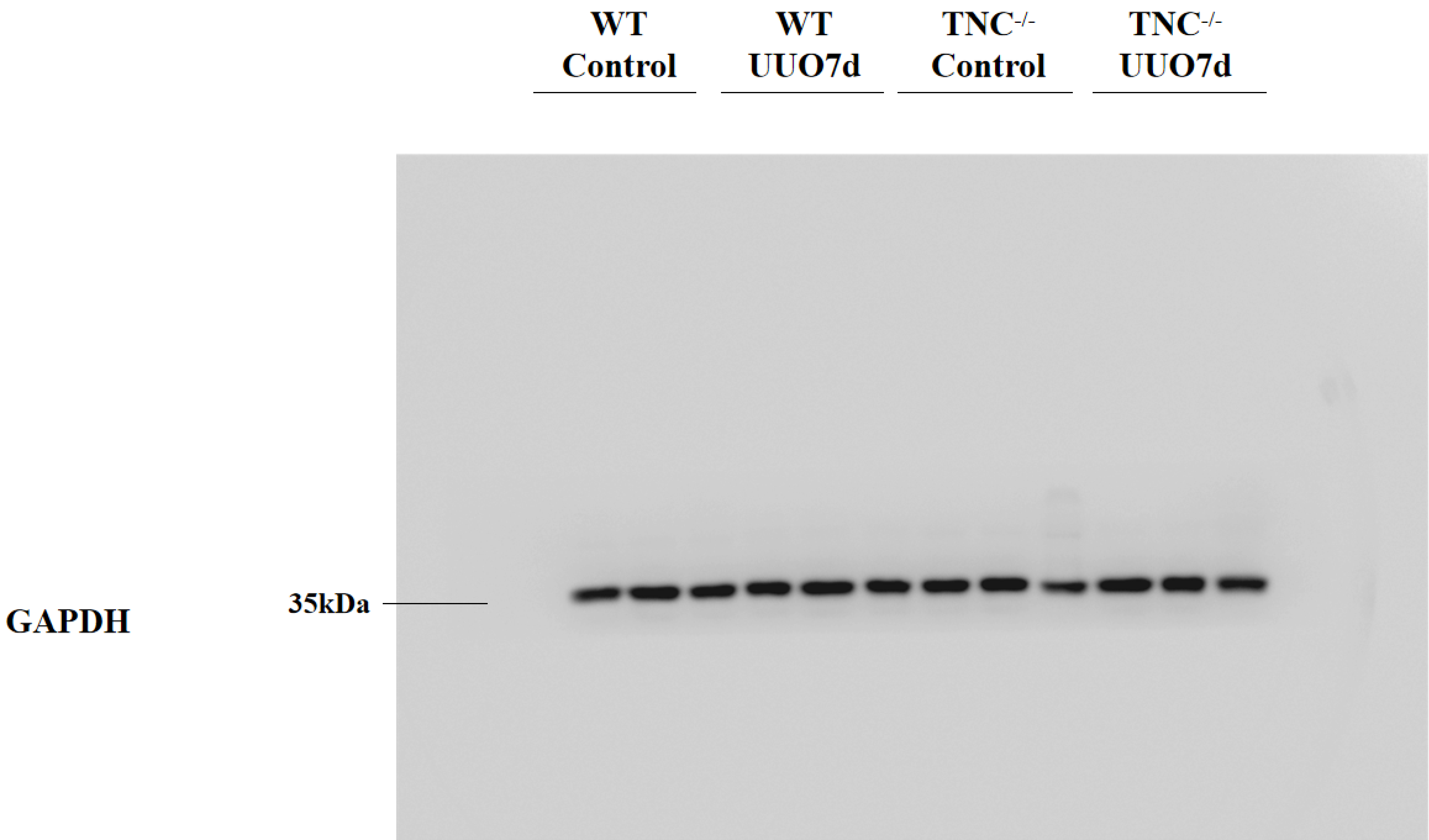
WT Control
WT UUO7d
TNC-/- Control
TNC-/- UUO7d
GAPDH
35kDa

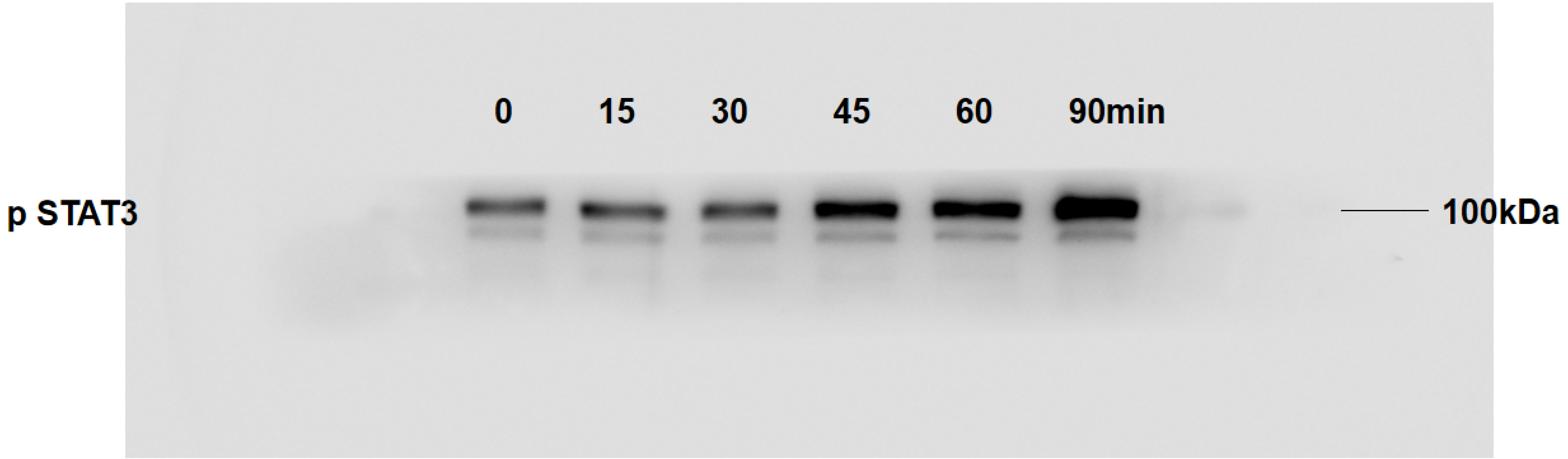
0
15
30
45
60
90min
p STAT3
100kDa

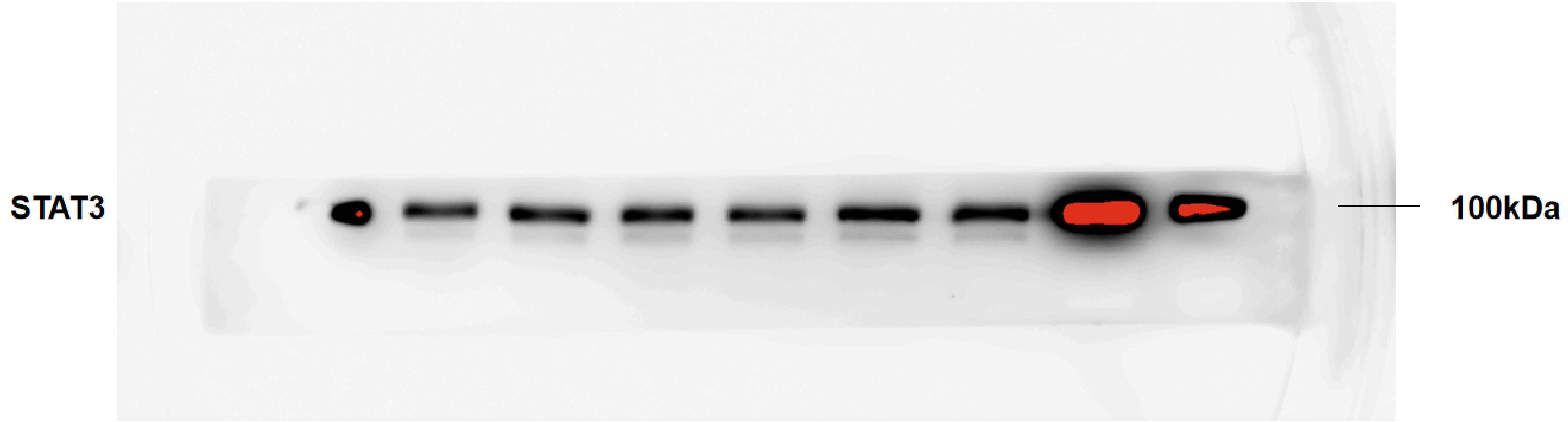
STAT3
100kDa

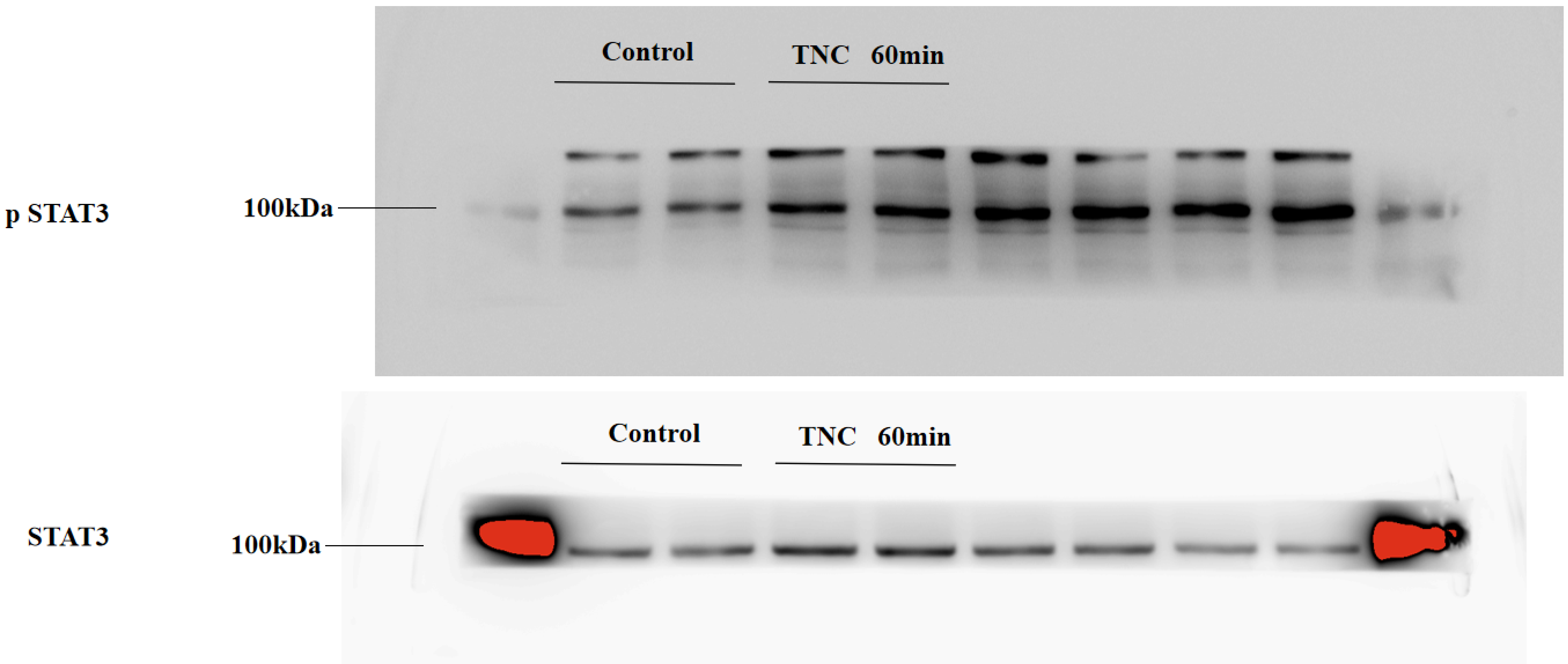
Control
TNC 60min
p STAT3
100kDa
Control
TNC 60min
STAT3
100kDa

p EGFR 180kDa

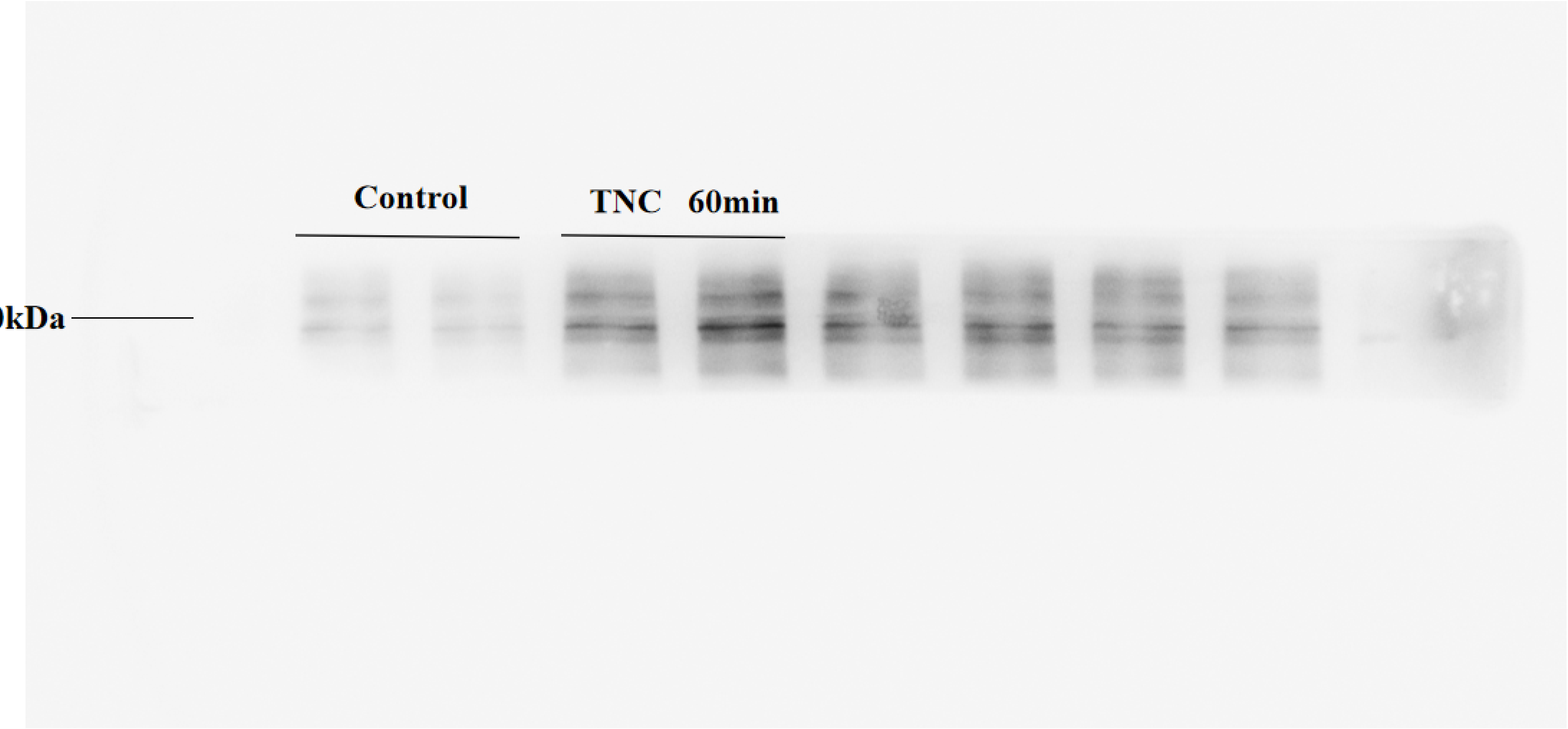

EGFR 180kDa

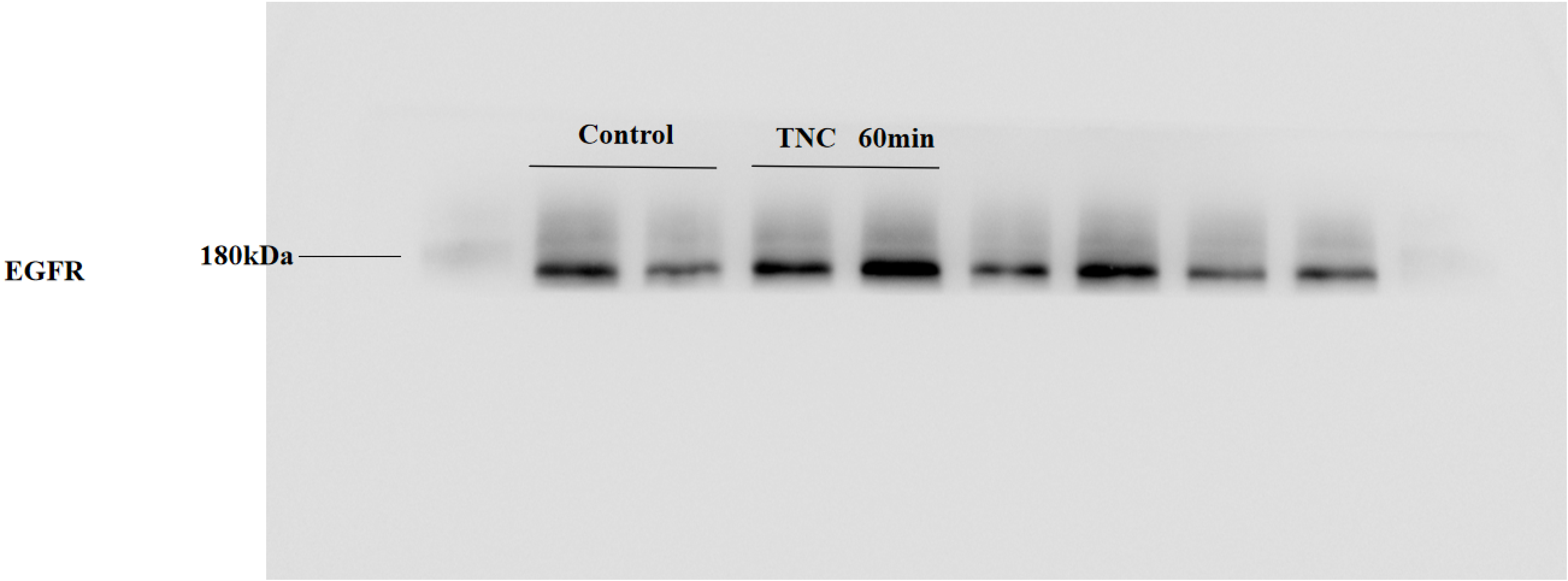

Supplement: Supplementary file 11 — Original Data File [file 41419_2022_5496_MOESM11_ESM.pdf]
